# 3D Bioprinting in Anterior Segment Ocular Tissue Engineering: Advances, Challenges, and Translational Pathways—A Systematic Review

**DOI:** 10.3390/cells15181686

**Published:** 2026-09-17

**Authors:** Konstantinos Terlemes, Mikaela Shakalli, Sotiria Palioura

**Affiliations:** 1 School of Medicine, Faculty of Health Sciences, Aristotle University of Thessaloniki, 54124 Thessaloniki, Greece; terlemesk@gmail.com (K.T.); mikaelashakalli@gmail.com (M.S.); 2 Department of Ophthalmology, Bascom Palmer Eye Institute, University of Miami Miller School of Medicine, Miami, FL 33136, USA

**Keywords:** bioprinting, cornea, conjunctiva, anterior eye segment, lacrimal apparatus, ocular surface, limbal stem cells, tissue engineering, systematic review, reporting standards

## Abstract

**Highlights:**

**What are the main findings?**

- A total of 38 of 71 studies target the corneal stroma, three the endothelium, and none the iris, trabecular meshwork or ciliary body.
- Forty studies are in vitro only, and no reported follow-up exceeds 90 days.

**What are the implications of the main findings?**

- Only 36 of 61 corneal studies state a transparency wavelength and 26 report mechanics with units, so constructs cannot be compared.
- We propose a minimum reporting set to make future studies comparable.

**Abstract:**

Bioprinting has been proposed for nearly every structure of the anterior eye, but the effort is unevenly spent. We systematically reviewed 71 experimental studies of bioprinting across the anterior segment: cornea, conjunctiva, sclera, lens, lacrimal gland and eyelid. Most work targets the corneal stroma. The endothelium, the layer most often replaced surgically and the first to reach a clinical trial, accounts for the fewest preclinical studies. Beyond the cornea, printed constructs are mostly disease models rather than implants, and the iris, trabecular meshwork and ciliary body remain untouched. Preclinical testing remains short-term and confined to small animals. Studies measure the same properties in different ways, so constructs cannot be compared across laboratories. We map what has been printed for each tissue and how far each construct stands from the clinic, and propose a minimum reporting set to make the next generation of studies comparable.

## 1. Introduction

### 1.1. Burden of Anterior Segment Disorders and the Promise of 3D Bioprinting

Corneal disease and anterior segment disorders are major causes of visual impairment worldwide. An estimated 12.7 million people await corneal transplantation, and only about one in seventy receives a graft [1,2,3]. Advances in eye banking have not closed this gap. Decellularized scaffolds, stem cell transplantation and synthetic keratoprostheses each address part of the problem, yet remain constrained by donor supply, immune rejection, or incomplete restoration of native function [4].

Three-dimensional (3D) bioprinting offers a route around these constraints. Layer-by-layer deposition of living cells and biomaterials can generate patient-specific constructs that reproduce the architecture, transparency and function of ocular tissue [5,6,7]. Proof-of-concept constructs now exist for the cornea, conjunctiva, sclera, lens and lacrimal gland [8,9,10,11,12].

Corneal bioprinting is already well reviewed [5,6], but existing reviews are cornea-centered, organized by bioink or printing modality, and rarely assess the anterior segment as a whole or grade how close each tissue is to the clinic.

This review spans the anterior segment and ocular adnexa, from the cornea to the lens capsule, lacrimal gland and tear-film system and has three objectives. The first is to establish how bioprinting maturity varies across the anterior segment, separating tissues advancing toward a clinical product from those still at in vitro proof of concept. The second is to identify the tissue-specific factors that explain that heterogeneity: geometry, cell sourcing, vascularization and the functional endpoint each tissue must meet. The third is to define the barriers that must be cleared to move these constructs from bench to bedside.

### 1.2. Bioprinting Fundamentals

In this review, 3D bioprinting means the computer-controlled additive fabrication of constructs intended to replace, regenerate, repair, or model living ocular tissue. The definition turns on the fabrication method and on that tissue-directed purpose; the presence of cells at the moment of printing does not by itself determine eligibility. Three categories follow, and they are kept distinct throughout: constructs in which cells are suspended in the bioink and deposited during fabrication; printed scaffolds cellularized after fabrication; and a small number of acellular constructs printed for direct surgical repair, admitted only where they carry a defined tissue-repair function. These categories are distinguished wherever the distinction affects how an outcome should be read. So defined, 3D bioprinting applies additive manufacturing to cell-compatible biomaterials, enabling precise spatial organization of multiple components within tissue-like constructs [13].

**Figure 1:**
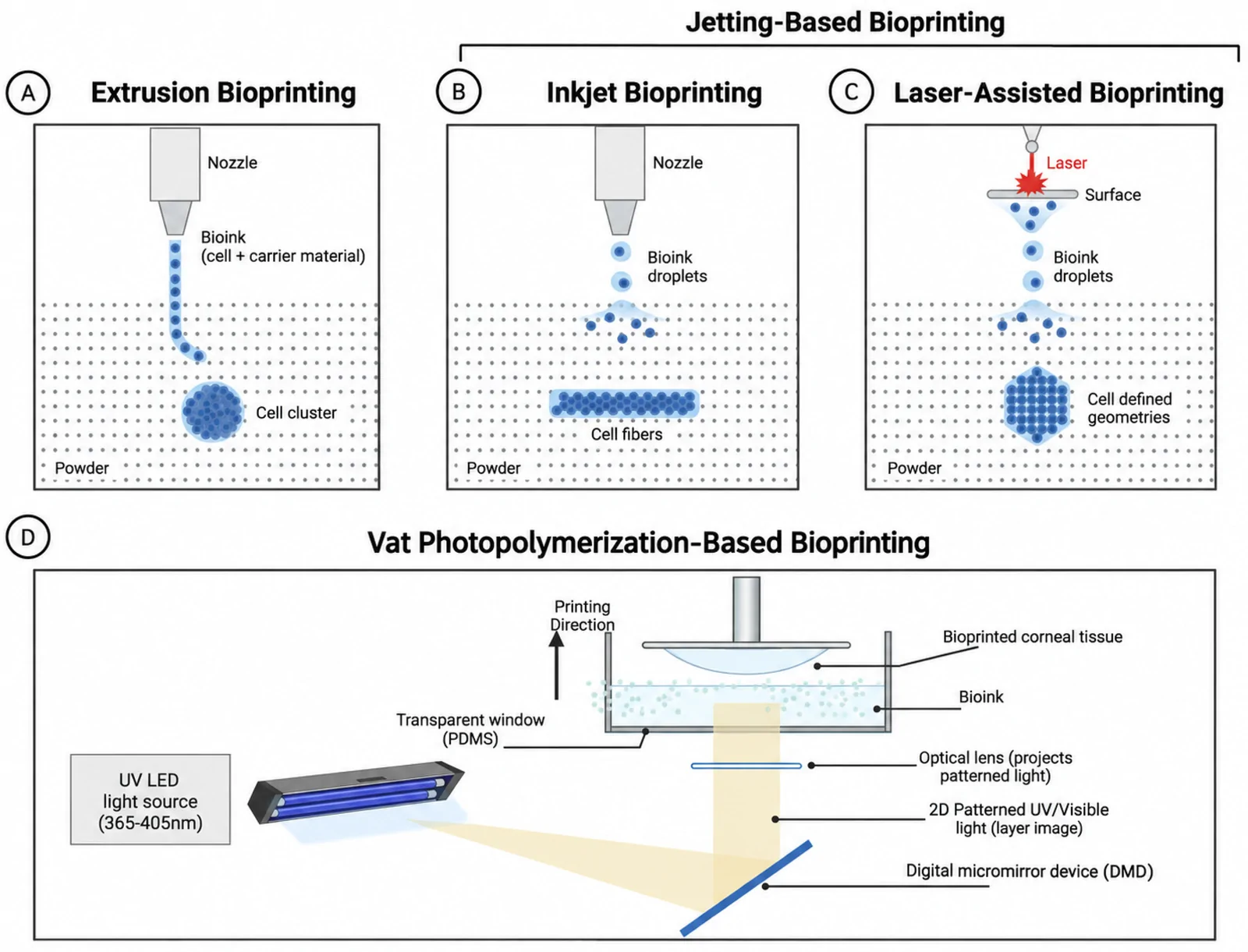
Principal 3D bioprinting modalities, grouped by ASTM-aligned family, with application to ocular constructs such as corneal tissue. (**A**) Extrusion-based bioprinting: bioink-containing cells and carrier material are deposited as a continuous filament through a nozzle onto a powder substrate, building a cell cluster. (**B**,**C**) Jetting-based bioprinting. (**B**) Inkjet: bioink droplets are ejected from a nozzle onto the powder substrate to form cell fibers. (**C**) Laser-assisted: a laser pulse propels bioink droplets from a donor surface onto the powder substrate to create defined cell geometries. (**D**) Vat photopolymerization-based bioprinting: a UV LED source (365–405 nm) illuminates a digital micromirror device (DMD) that generates a two-dimensional patterned light image, which an optical lens projects through a transparent polydimethylsiloxane (PDMS) window to polymerize the bioink layer by layer while the build platform moves upward, producing a bioprinted corneal tissue. Created with BioRender.com.

Modality nomenclature here follows the ASTM-aligned classification of additive manufacturing processes. Three families dominate ocular work: extrusion-based, jetting-based (droplet ejection onto a substrate, comprising inkjet and laser-assisted printing) and vat photopolymerization-based bioprinting. Several labels used in the primary literature belong within these families: digital light processing and stereolithography are vat photopolymerization; drop-on-demand and spray deposition are jetting-based; and handheld, biopen and in situ devices are extrusion-based variants distinguished by where printing occurs rather than by mechanism. Hybrid and multi-nozzle systems combine two families, and 4D approaches add a stimulus-responsive material to one of them; both are identified as such wherever the underlying studies are discussed. Extrusion-based bioprinting deposits continuous hydrogel filaments through a nozzle, tolerates high-viscosity, cell-dense bioinks, and readily produces curved corneal constructs, making it the workhorse for stromal and multilayer models; in situ variants allow direct deposition into surgical defects [14,15,16,17]. Within the jetting-based family, inkjet bioprinting dispenses picoliter droplets at high resolution but requires low-viscosity inks and generally yields thinner constructs [18]. Laser-assisted bioprinting, the nozzle-free member of the same jetting family, achieves micron-scale precision at the cost of throughput [5]. Vat photopolymerization-based bioprinting, including digital light processing (DLP) and volumetric bioprinting, cures entire layers or volumes within seconds, enabling rapid fabrication of transparent, anatomically complex tissues [19,20]. These modalities are complementary, and the choice for a given construct follows its geometry, resolution needs and bioink rheology (Figure 1).

Bioink selection is equally determinative. Natural polymers (collagen, gelatin, hyaluronic acid, fibrin, alginate) resemble extracellular matrix (ECM) and support adhesion and differentiation, and collagen underpins corneal transparency and strength, but pure natural gels are usually too weak to hold shape without crosslinking [21,22]. Synthetic polymers such as gelatin methacryloyl (GelMA), poly(ethylene glycol) diacrylate (PEGDA) and 2-hydroxyethyl methacrylate (HEMA)-based hydrogels give tunable stiffness, degradation and print fidelity but lack biological signals [23]. Hybrids combine the two: dECM blended with GelMA preserves biochemical cues while holding shape and clarity [24]. For the cornea, bioinks must reach > 80–90% transmission while withstanding intraocular pressure, usually through double-network or dual-crosslinking designs [22,25,26].

Cell sources map onto the target layer. Limbal epithelial stem cells (LSCs), including those derived from embryonic and induced pluripotent stem cells (iPSCs), are the physiological choice for epithelium, while mature corneal epithelial cells have been sprayed or patched to speed wound closure [27,28,29,30]. The stroma uses keratocytes, which dedifferentiate on expansion, so groups substitute keratocyte-differentiated mesenchymal stem cells or stromal cell lines in GelMA or collagen [31,32,33]. For endothelium, primary and iPSC-derived endothelial cells are printed in fast-gelling hydrogels, including onto denuded Descemet’s membrane [34,35,36,37].

After printing, constructs are crosslinked and matured in culture, and both steps shape the final transparency, stiffness and cell phenotype [31,38,39].

Each modality imposes its own set of process parameters, and these govern cell survival, phenotype retention and print fidelity independently of bioink chemistry. Reported viability figures are interpretable only alongside the printing conditions that produced them. The parameters that matter most within each family, and their consequences for ocular constructs, are set out below [40].

#### 1.2.1. Process Parameters in Extrusion-Based Bioprinting

Cell damage in extrusion-based bioprinting is dominated by wall shear stress generated as the bioink passes through the nozzle. Shear stress rises with dispensing pressure and bioink viscosity and falls as nozzle diameter increases, placing resolution and viability in direct competition. Nozzle geometry modifies the same relationship: conical nozzles generate lower peak shear than cylindrical needles of equal outlet diameter, and shorter nozzles reduce the time cells spend under load. Exposure duration compounds magnitude, so slow deposition through a fine nozzle is more damaging than the peak shear value alone suggests [40]. The crosslinking strategy then determines the mechanical and optical fate of the construct. Ionic crosslinking of alginate is rapid and cytocompatible but yields weak, hydrolytically unstable gels, whereas photo-crosslinking of GelMA gives tunable stiffness and the transparency required for corneal work at the cost of the photoinitiator and light exposure. For corneal stroma, the usable window is narrow: crosslinking must be sufficient to hold curvature and resist intraocular pressure without producing the substrate stiffness that drives keratocyte activation [40].

#### 1.2.2. Process Parameters in Jetting-Based Bioprinting

Cells in jetting-based bioprinting experience two distinct insults: shear during droplet formation within the nozzle or chamber, and impact stress as the droplet meets the substrate. Shear during droplet formation has been quantified most directly in microvalve-based systems: Blaeser et al. controlled shear at the nozzle of a microvalve printer and found that viability and subsequent proliferative capacity were largely preserved below approximately 5 kPa, declining progressively above that level [41,42]. Thermal printheads add a brief, localized temperature rise, although the heated volume is small and the exposure lasts microseconds. Xu et al. showed that mammalian cells survive thermal inkjet deposition with viability close to unprinted controls [43]. Droplet impact velocity and droplet volume are the two parameters most consistently linked to loss of viability. Ng et al. dispensed sub-nanoliter cell-laden droplets from a thermal inkjet system and identified both as significant determinants of the viability and proliferation of printed cells; raising the cell concentration slowed impact velocity, which improved viability and limited droplet splashing [44]. Impact velocity scales with actuation voltage or pressure, so gains in placement accuracy are again paid for in cell stress [45]. Droplet volume and nozzle diameter set the achievable resolution and also determine how many cells each droplet carries; smaller droplets improve resolution but raise clogging risk and sensitivity to cell sedimentation in the reservoir. Bioink properties constrain the process throughout: jetting demands low viscosity, in the low mPa·s range for inkjet, together with surface tension within a narrow band, which limits polymer concentration and with it the mechanical strength of the printed construct [42,45]. Laser-assisted printing eliminates clogging and nozzle shear but substitutes laser fluence, ribbon coating thickness and donor-layer viscosity as the determinants of droplet size and cell survival [40].

#### 1.2.3. Process Parameters in Vat Photopolymerization-Based Bioprinting

Viability in vat photopolymerization depends on photochemistry more than on mechanics. Photoinitiator identity and concentration set both crosslinking rate and cytotoxicity. Irgacure 2959 is water-soluble but absorbs poorly at wavelengths regarded as benign to cells, which forces long exposure times, whereas lithium phenyl-2,4,6-trimethylbenzoylphosphinate (LAP) absorbs efficiently at 405 nm and polymerizes faster at lower concentration, making it the default initiator for cell-laden work [46]. Cytotoxicity is a property of initiator and light in combination, not of the initiator alone: LAP concentrations tolerated in the dark became cytotoxic to mammalian cells under 405 nm exposure, while remaining non-mutagenic in bacterial reverse mutation assays [47]. Wavelength selection carries particular weight for ocular tissue, since 365 nm ultraviolet crosslinking is poorly suited to cells of the anterior segment and visible-light initiating systems achieve comparable print fidelity in gelatin-based bioinks without ultraviolet exposure [48]. Light intensity and total dose govern cure depth and interlayer adhesion. Excessive dose overcures the construct, raising stiffness and reducing transparency, while insufficient dose leaves a weakly crosslinked construct that deforms after printing. Photon penetration falls with depth and with the light scattering produced by dense cell suspensions, so thicker or more cellular corneal constructs require dose compensation that itself increases the radical burden on the encapsulated cells [40,48].

### 1.3. Advanced Bioprinting Strategies for the Corneal Microstructure

The cornea is geometrically simple, a transparent avascular dome roughly half a millimeter thick, but its optical and mechanical function rests on a microstructure that conventional layer-by-layer printing does not reproduce. The stroma consists of stacked lamellae oriented at near-orthogonal angles, each assembled from collagen fibrils of uniform narrow diameter held at regular interfibrillar spacing [26]. This organization, and not composition alone, generates transparency and the anisotropic stiffness that sustains curvature under intraocular pressure. Reproducing it requires control of fibril orientation, dome curvature and layer-to-layer registration at a scale finer than the printed filament itself, which is why several strategies have been developed specifically to add sub-filament order to printed corneal constructs.

The first exploits the printing process itself as an alignment tool. Extrusional flow through a fine nozzle imposes shear that orients collagen fibrils along the print path, so depositing successive layers at rotated angles reconstructs the orthogonal lamellar stack. Kim et al. applied this principle to corneal stroma and obtained constructs in which both fibril orientation and keratocyte elongation followed the print direction, with transparency approaching that of native tissue [49]. Print-path design in this approach carries structural information, and the same nozzle shear that threatens viability becomes the mechanism that builds architecture.

The second addresses curvature and thickness. Vat photopolymerization cures a whole layer at once, decoupling geometric complexity from print time, and has been used to fabricate curved corneal constructs with defined thickness profiles by stereolithography [50]. Volumetric bioprinting extends this further by projecting tomographic visible-light patterns into a rotating vat, forming centimeter-scale constructs in seconds with viability above 85% [51]. For the cornea, this removes the horizontal layer interfaces that scatter incident light, and the short fabrication time limits the radical exposure.

The third solves the mechanical problem of printing weak, physiologically formulated inks. Embedded printing into a thermoreversible support bath allows low-viscosity collagen to be deposited at neutral pH and body temperature while the bath holds the geometry until crosslinking is complete, achieving microscale features in collagen that cannot support their own weight in air [20]. The approach is directly relevant to corneal work, where the inks with the best biological credentials are the least printable.

The fourth targets stratification. Laser-assisted printing has been used to deposit limbal epithelial stem cells and stromal cells as discrete layers with defined cell-type boundaries [28], and dual-crosslinking hybrid inks decouple print fidelity from final transparency, allowing a construct to be shaped in a stiff transient state and matured into a clear one [52]. Combining these with multi-head extrusion offers a route to epithelium, stroma and endothelium in a single build, though no study has yet demonstrated all three layers with functional endpoints in vivo. The strategies in this section are best read as capability demonstrations.

## 2. Materials and Methods

### 2.1. Data Sources and Search Strategy

This systematic review followed the Preferred Reporting Items for Systematic Reviews and Meta-Analyses (PRISMA) 2020 statement [53]; the completed checklist is provided as Table S1. The protocol was prospectively registered in the PROSPERO database (International Prospective Register of Systematic Reviews) under the reference number CRD420251113745. We searched PubMed and Scopus from inception through August 2025 using a predefined Boolean strategy combining bioprinting terms with anterior segment structures: (“3D bioprinting” OR “bioprinting” OR “three-dimensional bioprinting”) AND (“cornea” OR “corneal” OR “conjunctiva” OR “conjunctival” OR “limbus” OR “limbal” OR “sclera” OR “scleral” OR “iris” OR “trabecular meshwork” OR “anterior chamber angle” OR “lens” OR “crystalline lens” OR “ocular surface” OR “anterior segment” OR “lacrimal gland” OR “lacrimal system” OR “tear duct” OR “tear film” OR “dry eye”). We also performed backward citation screening of included full texts, and a search update in August 2026 added adnexa-specific terms (“eyelid”, “tarsal plate”, “meibomian gland”, “nasolacrimal”, “canaliculus”) beyond the original search terms and screened against the same eligibility criteria. Searches were limited to English-language publications.

### 2.2. Study Selection

Duplicate records were removed in Zotero (version 7.0.24, Corporation for Digital Scholarship, Vienna, VA, USA). Two independent reviewers (KT, MS) then screened records against prespecified criteria. Records passing title and abstract screening proceeded to full-text assessment, with discrepancies resolved by the third reviewer (SP). We included original experimental studies (in vitro, ex vivo, or in vivo animal) that used 3D bioprinting to fabricate constructs targeting anterior segment ocular tissues and reported outcomes relevant to tissue engineering, regeneration, disease modeling, or drug delivery. We excluded reviews, editorials, conference abstracts without full text, posterior segment applications (retina/choroid/vitreous), non-ophthalmic uses, purely acellular printing with no tissue-repair or tissue-modeling function (e.g., plastic scaffolds or instrumentation), and computational work without experimental validation. Eligibility was then assessed against three sequential tests. First is fabrication: the construct had to be produced by 3D printing or another digitally controlled additive manufacturing process. Studies in which the scaffold was formed by electrospinning, molding, casting, or solvent evaporation were excluded even where cells were subsequently applied. Second is cellular component and intent: the study had to fall into one of the three categories defined in Section 1.2 (cells present during printing, post-print cellularization, or acellular constructs printed for direct surgical repair), and the printed object had to be intended to replace, regenerate, repair, or model ocular tissue. Constructs whose function was purely mechanical or pharmacological, such as contact lenses, drug-eluting inserts, symblepharon rings, and bypass tubes, were excluded even when cytocompatibility had been demonstrated. Third is tissue specificity: the study had to target a defined anterior segment ocular tissue, and studies presenting the cornea as one illustrative target among several unrelated tissues were excluded. No restrictions were placed on geography, sample size, or follow-up duration.

### 2.3. Data Extraction and Synthesis

Two reviewers (KT, MS) independently extracted data using a standardized form. Information collected included study identification (authors, year, journal), targeted tissue or corneal layer, main application (e.g., regeneration, disease modeling, drug delivery), and technical parameters: bioink composition (e.g., collagen, GelMA, alginate, fibrin, hyaluronic acid, decellularized ECM), crosslinking method, bioprinting technique classified by ASTM-aligned family (extrusion-based; jetting-based, comprising inkjet and laser-assisted; and vat photopolymerization-based, including DLP), with hybrid and multi-nozzle systems recorded as combinations, and digital modeling workflow.

Each study was additionally classified by its intended application into one of two translational pathways: (1) implantation and regeneration, comprising constructs intended as tissue substitutes or as vehicles for regenerative cell or factor delivery; and (2) in vitro disease modeling and drug screening, comprising constructs intended as laboratory models and not for implantation. Classification followed each study’s stated purpose and primary evaluation, not merely whether an animal model was used, so that an in vitro prototype of an implantable construct was assigned to the first pathway and an advanced disease model that was never implanted to the second. The two pathways are evaluated against different endpoints and are synthesized separately.

Biological and functional outcomes recorded included cell viability, phenotype/marker expression, transparency, biomechanical properties (e.g., modulus, tensile strength, degradation), and in vivo performance where applicable. Printing parameters such as nozzle gauge, extrusion pressure, layer thickness, speed, and temperature were noted when reported. A single reporting convention was applied to the three principal outcomes. Cell viability was recorded as a percentage with the assay and the timepoint. Optical transparency was recorded as a percentage with the wavelength or wavelength range at which it was measured. Biomechanical performance was recorded with the modulus type named and the value given with units, keeping compressive, tensile and storage moduli distinct. Outcomes a study did not measure were recorded as N/R, and qualitative descriptors were not carried into the tables.

Given the heterogeneity of study designs, bioprinting methods, and outcome measures, quantitative meta-analysis was not feasible. Results were synthesized narratively and summarized in comparative tables, with greater detail for corneal studies, which predominate.

### 2.4. Appraisal of Methodological Quality

A formal risk-of-bias assessment was not performed. Validated instruments exist for defined study types, but no single instrument covers the range of designs included here, most of which are early-stage in vitro feasibility or material-characterization studies without standardized endpoints. Applying a tool to the in vivo subset alone would give a partial appraisal that is not comparable across the review. We appraised all studies for reproducibility (reporting of print parameters and bioink composition), translational relevance (phenotype maintenance, biocompatibility, and in vivo performance) and completeness of reporting, which better reflects the developmental stage of ocular bioprinting research. Two extraction conventions applied throughout. Transparency was recorded as quantitative only where a study reported a numeric percentage together with a numeric wavelength or wavelength range; values presented only as figure curves, with no number in the text, a table or a figure annotation, were recorded as assessed without an extractable value. Where one research group published overlapping work from a single experimental cohort in more than one publication, the reports were cited together and counted as one study.

## 3. Results

### 3.1. Search Results and Study Selection

**Figure 2:**
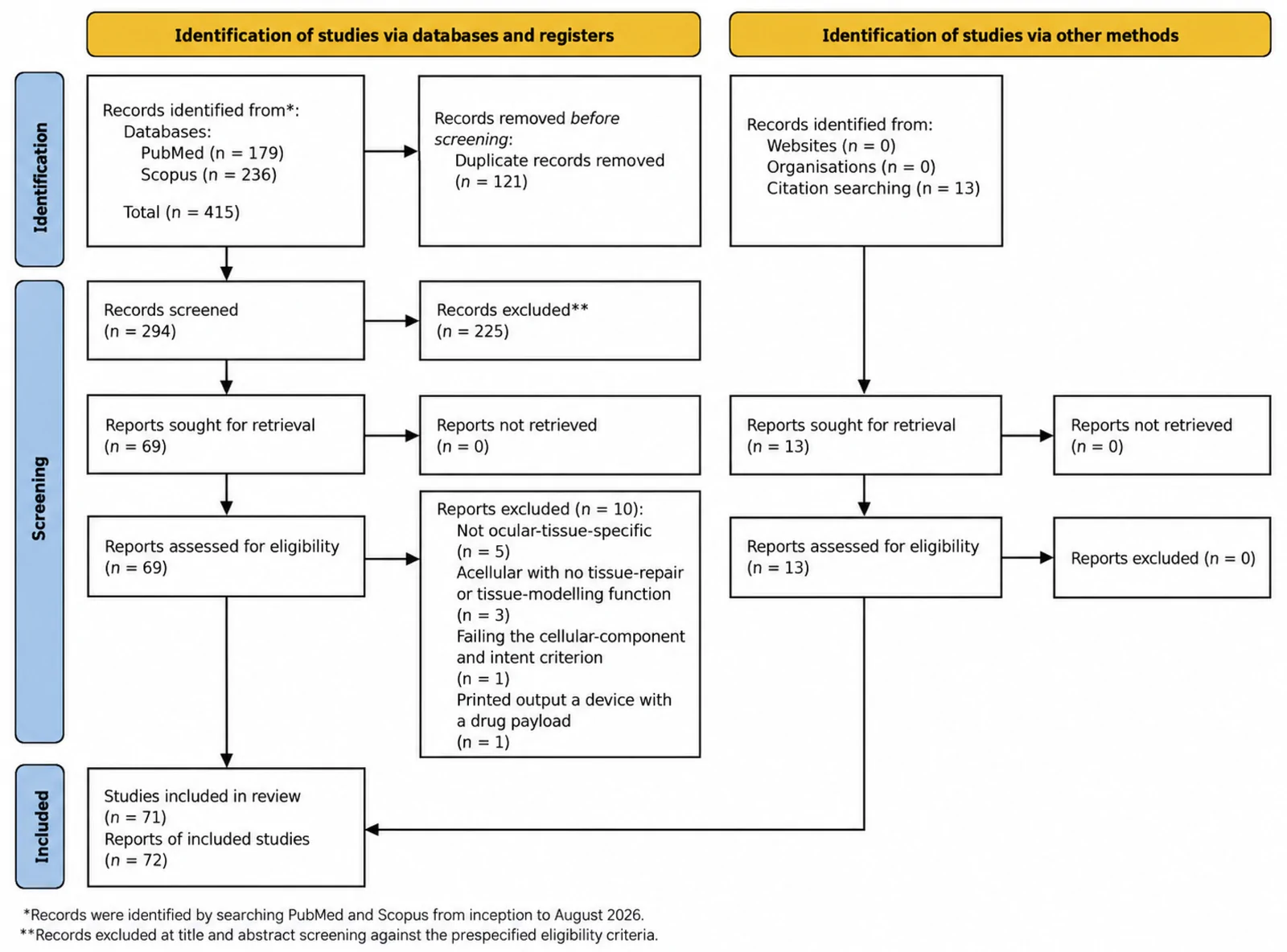
PRISMA flow diagram for study selection in the systematic review. The diagram illustrates the identification, screening, eligibility assessment, and inclusion of peer-reviewed studies on 3D bioprinting for anterior segment ocular tissues. Databases searched: PubMed (*n* = 179) and Scopus (*n* = 236). Records screened after duplicate removal (*n* = 294); full-text articles assessed (*n* = 69). Exclusions: duplicates (*n* = 121); irrelevant records (*n* = 225); full-text exclusions (*n* = 10 total): not ocular-tissue-specific (*n* = 5), acellular with no tissue-repair or tissue-modeling function (*n* = 3), failing the cellular-component and intent criterion (*n* = 1), printed output a device with a drug payload (*n* = 1). Backward citation searching: (*n* = 13). Reports of included studies: 72; studies included in the review: 71 (two reports describe the same study).

The database search identified 415 records (PubMed, *n* = 179; Scopus, *n* = 236). After removal of 121 duplicates, 294 records were screened by title and abstract, with 225 excluded as irrelevant. A total of 69 full-text articles were assessed for eligibility, of which 10 were excluded (five not specific to ocular tissue, three acellular with no tissue-repair or tissue-modeling function, one failing the cellular-component and intent criterion, and one whose printed output was a device with a drug payload). An additional thirteen reports were identified through citation searching, all of which were eligible. In total, 72 reports met the inclusion criteria; two of these (Kim et al., 2018a, 2019a) [54,55] described a single experimental cohort and were counted as one study, giving 71 included studies (Figure 2, PRISMA flow diagram).

### 3.2. Cornea

Corneal tissue has been the primary focus of ophthalmic bioprinting (61 of 71 included studies), reflecting its transparency and the demand for transplantable grafts. Work has targeted individual layers (epithelium, stroma, endothelium) and multilayer constructs aiming at full-thickness replacement. We synthesize the results by layer, then multilayer and specialized applications; all included corneal studies and their extracted parameters are given in Table S2.

#### 3.2.1. Corneal Epithelium

Ten included studies targeted the corneal epithelium (Table S2). Every reported viability figure was above 80%, and most exceeded 90% [7,8,27,35,56,57,58,59,60]; Xie et al. showed that the printing process itself reduces viability: cell survival fell from about 98% at low spray pressure to 88% at 138 kPa (20 psi) [58]. Transparency was measured in only two studies [35,57], and five progressed to in vivo testing in rodent, rabbit or porcine ocular-surface injury models [27,56,58,59,60]. Cell sourcing shifted over the decade, from mature corneal epithelial cells early on to LSCs or pluripotent-derived limbal subpopulations from 2024 [7,60,61].

Four studies illustrate the range. Wu et al. showed post-print softening matters: partially dissolving alginate from a gelatin–alginate–collagen bioink gave a looser network with better spreading, proliferation and cytokeratin-3 expression [35]. Zhong et al. showed the bioink can set stem-cell state, with limbal stem cells staying proliferative in GelMA but entering reversible quiescence in hyaluronic acid glycidyl methacrylate, letting active and quiescent niches be built into one construct [8]. Wang et al. addressed delivery, printing limbal stem cells in a thermosensitive chitosan hydrogel that conforms to an irregular wound bed and improved healing in rat alkali-burn and diabetic rabbit models [27,56]. Shang et al. extended this to alkali-burned Bama piglet corneas, whose structure resembles the human cornea, with single-cell sequencing showing more limbal stem cells in treated tissue [60].

The consistent limitation across this layer is stratification. Native corneal epithelium comprises five to seven cell layers, whereas the printed constructs in this review generally formed a monolayer at the point of printing, with multilayering achieved only through subsequent air–liquid interface culture [28]. No included epithelial study reported a fully stratified, terminally differentiated epithelium directly from the printer. Viability, marker expression and surgical handling are solved problems for this layer, but architecture is not.

#### 3.2.2. Corneal Stroma

Thirty-eight included studies addressed the corneal stroma, by far the most studied layer (Table S2). Post-print viability was mostly above 85%, with no dependence on printing modality [19,24,31,50,62,63,64,65,66,67,68,69]. Quantified transparency spanned 48.4% to 99.5%, a range set by deliberate parameter extremes, not inconsistency between studies. Ten studies reached in vivo implantation, nine in rabbits (one of which also used a mouse model) and one in rats, and three were tested ex vivo in porcine corneas [19,33,49,54,55,64,67,68,70,71,72,73,74,75]. Four did not report viability and six did not assess transparency. Three targets recur: transparency, suture-adequate strength, and suppression of the keratocyte-to-fibroblast transition that produces haze.

Bioink composition was the principal experimental variable. Isaacson et al. printed the first stromal equivalent, human keratocytes in alginate-methacrylated collagen within a sacrificial gelatin support, retaining keratocan and collagen-I expression [31]. Tissue-specific matrix proved advantageous: Kim et al. (2018, 2019) developed a porcine corneal dECM bioink transmitting > 75% across 300–700 nm that integrated into rabbit corneas with minimal immune response and supported lumican and keratocan expression [49,54,55]. GelMA has been the most common synthetic alternative as a moderately stiff formulation balanced clarity against cell support, reaching a compressive modulus near 20 kPa against 27 kPa for native cornea [62]. Hybrid dECM–GelMA and matrix–silk fibroin systems combined biochemical cues with print fidelity and strength [24,52].

Composition and micro-architecture produce a trade-off that three studies quantified. Gao et al. varied collagen concentration and fiber alignment and found transmittance falling from 89.8% at 700 nm (0.8% collagen) to 48.4% at 4%, showing that clarity and mechanical strength cannot be maximized together in a single-component collagen ink [39]. Puistola et al. showed the same effect from cell loading, transmittance falling to 67–84% once cells were present [66]. Controlled shear during extrusion aligned collagen fibrils along the print path, reproducing native lamellar organization and improving both strength and transparency, since aligned fibrils scatter less light [49,55]. Curvature also mattered: Xu et al. showed convex, dome-shaped implants integrated better in rabbit corneas, with less fibrosis and >85% transmission (400–700 nm), than flat constructs of identical composition [33].

Recent work has moved beyond structural replacement. Choi et al. cultured printed human stromal cells for four weeks and saw downregulation of fibrotic markers, suggesting the printed environment itself may suppress scarring [32]. Mörö et al. co-printed iPSC-derived peripheral neurons with keratocytes, with neurites extending through a construct transmitting 94.1% at 700 nm, partially reconstituting a corneal nerve plexus [64]. Kethiri et al. delivered a hyaluronic acid bioink to rat stromal pockets, comparing injectable and bioprinted formats carrying adipose stem cell-derived keratocytes; bioprinted constructs stayed transparent and integrated with host stroma [75]. Three limitations persist. Long-term optical stability is largely untested: the longest in vivo follow-up was 90 days [74]. Most hydrogel constructs remain softer than donor cornea, which bears on suturing and healing. And clinical processing can degrade performance: Yoon et al. found transparency at 550 nm fell after thermal gelation and terminal sterilization [76].

#### 3.2.3. Corneal Endothelium

Only three included studies targeted the corneal endothelium, the thin (5–10 μm) monolayer of hexagonal cells that maintains stromal deturgescence and whose loss causes edema and opacity in Fuchs dystrophy and bullous keratopathy [37,77,78]. All three printed a confluent monolayer expressing tight-junction and pump markers, but they differ sharply in how far each was tested. Kim et al. bioprinted an approximately 8 mm endothelial graft of human corneal endothelial cells overexpressing ribonuclease 5, a factor known to enhance endothelial proliferation and survival, in a gelatin bioink onto lyophilized amniotic membrane; this is the only included endothelial construct implanted in vivo, and corneal clarity began to be restored from two weeks after transplantation in rabbits, with central corneal edema markedly less than controls at three and four weeks [77]. Grönroos et al. printed pluripotent stem cell-derived corneal endothelial cells in a hydrazone-crosslinked hyaluronic acid bioink, achieving approximately 92.5% viability at day 7 with native-like localization of ZO-1, Na+/K+-ATPase and CD166; the construct was validated ex vivo on denuded rat, porcine and human Descemet’s membrane but was not transplanted [37]. Overmass et al. printed human corneal endothelial cells in collagen type IV and reported 97.8 ± 0.5% transmittance across 400–700 nm, the highest mean transmittance reported for any construct in this review, with a tensile Young’s modulus of 89.5 ± 23.9 kPa against 49 ± 14 kPa in compression; testing was confined to in vitro [78].

Two observations follow. First, the endothelium is the least studied corneal layer, and the reporting gap is correspondingly wide: viability was not reported at all in one of the three studies, and transparency was quantified in only one. Second, no included study followed endothelial cell density or pump function beyond four weeks, and none assessed the fibrocellular overgrowth that limits conventional endothelial keratoplasty. The layer combines the most favorable geometry for bioprinting, a single monolayer on a defined basement membrane, with the thinnest evidence base in this review.

#### 3.2.4. Multiple Corneal Layers

**Figure 3:**
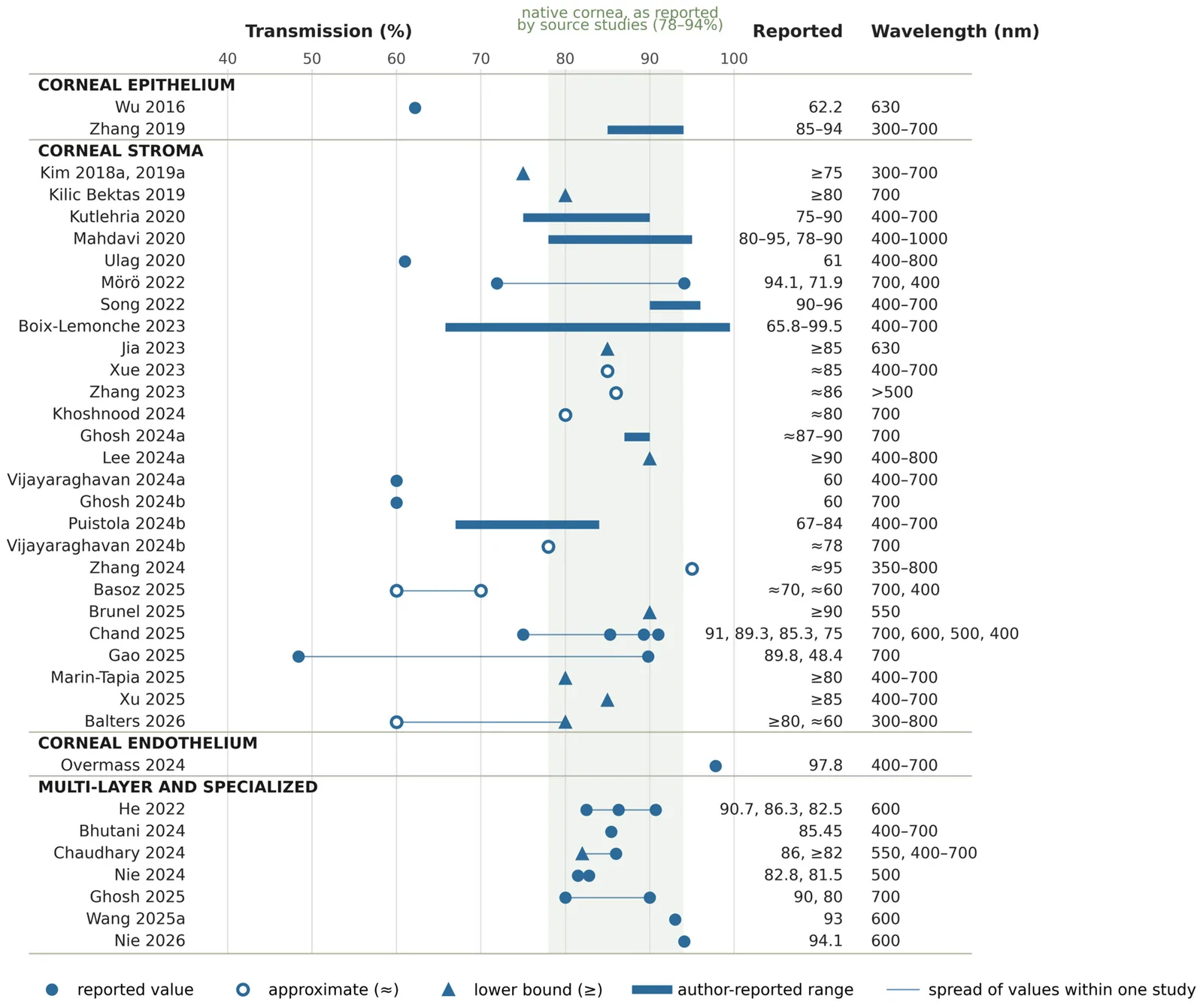
Reported light transmission of bioprinted corneal constructs, plotted at the wavelength each study reported. Each row is one reported measurement: a circle marks a single value, a triangle a lower bound (“≥X%”), and a bar the full range reported by the source study. The wavelength or range for each value is given in the right-hand column; values at different wavelengths are not interchangeable and are not pooled. A study reporting at more than one wavelength or formulation contributes more than one row. Rows are grouped by target corneal layer. The shaded band marks native human corneal transmittance, which is itself wavelength-dependent, rising from roughly 80% at 400 nm to above 90% beyond 500 nm [25,26]. Corneal studies that reported transparency only qualitatively are not plotted and are listed in Table S2. No interpolated, midpoint or otherwise derived value is shown: the dispersion of transmission values and of the wavelengths at which they were measured is itself the reason a quantitative synthesis of optical performance was not attempted. Two constructs exceed native corneal transmission as reported by their authors, which reflects measurement on thin acellular or hydrated samples, not superior optical performance.

Seven included studies printed constructs comprising more than one corneal layer (Table S2). Where transparency was quantified, values clustered between 81.5% and 94.1% at 500–600 nm (Figure 3), and four progressed to in vivo implantation in rabbit anterior lamellar keratoplasty or limbal stem cell deficiency models [29,30,79,80]. The key finding is uniform: no study printed a cell-laden construct spanning all three corneal layers. The cell-laden multilayer constructs all combined an epithelial layer with a stroma [28,29,30,79,81], and in the implanted models, the host’s own endothelium maintained deturgescence. Two constructs were printed cell-free but evaluated with cells: Nie et al. [80] implanted a growth-factor- and drug-loaded epithelial/stromal bilayer in rabbits and supported corneal cells on it in vitro, and Chaudhary et al., the only construct spanning three structural layers, printed an alginate/cellulose-nanofiber cornea seeded with cells afterwards (82–96% viability at day 7) and not implanted [82].

Sorkio et al. established the approach, using laser-assisted bioprinting to deposit a layer of human adipose stem cell-derived keratocytes overlaid with embryonic stem cell-derived LSCs in laminin and collagen I, and implanting the stromal constructs into organ-cultured porcine corneas; optical transparency was not assessed [28]. He et al. used DLP to fabricate an epithelial and stromal bilayer in one continuous process from a GelMA–PEGDA composite, reaching approximately 90% post-print viability and 82.5–90.7% transmittance at 600 nm, and the graft remained transparent in a rabbit lamellar keratoplasty model over at least eight weeks with host epithelium growing over the implant [30]. Wang et al. applied the same chemistry to the limbus, not the central cornea, printing corneal epithelial cells in the superficial layer and corneal stromal stem cells in a niche layer beneath, and transplanted the construct into a rabbit limbal stem cell deficiency model, where epithelial healing was faster and opacity and neovascularization were reduced relative to controls at four weeks [29].

Two studies then addressed the surgical problem that a printed graft must be fixed in place. Nie et al. [80] printed a gelatin/carbohydrazide-modified alginate bilayer carrying recombinant epidermal growth factor and trichostatin A; the graft was cell-free at implantation but supported corneal cell growth in vitro, and reached a tensile strength of 1.068 MPa with elongation over 400% and suture retention adequate for anterior lamellar keratoplasty; the scaffold promoted epithelial and stromal regeneration and limited scarring in rabbits. Nie et al. [79] removed sutures altogether, combining a DLP-printed GelMA and methacrylated chondroitin sulfate bilayer carrying corneal epithelial cells and corneal stromal stem cells with an in situ photocrosslinkable bioadhesive; the construct transmitted 94.1 ± 0.5% at 600 nm, degraded almost completely within two weeks of implantation, and corneal curvature returned to physiological levels by week four. Puistola et al. extended multi-material printing to a stroma with an overlying epithelium from human stem cell sources, with transparency measured across 400–700 nm and a storage modulus of 112.2 ± 6.6 Pa at 1 Hz [81].

#### 3.2.5. Specialized and Emerging Applications of Corneal Bioprinting

A further group used bioprinting to make discrete intrastromal implants and surgical patches (Table S2). Three printed lenticules or inlays for partial-thickness transplantation or refractive correction [19,70,83]. Xue et al. printed ultra-thin (≈150 μm) GelMA inlays matched to corneal curvature that stayed clear in rabbits for over two months [70]. Bhutani et al. printed HA-methacrylate/GelMA lenticules that supported epithelial overgrowth and stromal infiltration in vitro and were stiff enough for surgical handling [83]. Jia et al. shaped the lenticule to a refractive prescription (46 D, refractive index 1.37) and produced the intended refractive change in rabbits over four weeks via femtosecond laser-assisted intrastromal keratoplasty [19].

Two studies addressed how a construct reaches the stroma. Boix-Lemonche et al. printed thin nanocellulose scaffolds laden with adipose-, bone marrow- and corneal stroma-derived MSCs and placed them in femtosecond-laser intrastromal pockets in porcine corneas; constructs retained 65.8–99.5% transmittance, integrated with minimal inflammation, and released anti-angiogenic factors [73]. You et al. took the opposite approach, printing directly onto the eye with a handheld device that deposited a platelet lysate bioadhesive onto rabbit corneal perforations, sealing the defect without sutures and healing faster than cyanoacrylate glue [84].

Key studies from each of the five subsections above are compared in Table 1. A third approach printed acellular constructs cellularized afterwards. Ghosh et al. printed transparent adhesive patches from dopamine-conjugated silk fibroin and decellularized corneal matrix, reaching 90% transmittance at 700 nm for the hybrid and supporting >85% viability at fourteen days [85].

### 3.3. Anterior Segment Tissues Beyond the Cornea

Ten included studies targeted anterior segment tissues other than the cornea: three the conjunctiva, three the lacrimal gland, two the lens or posterior capsule, one the sclera, and one the eyelid/meibomian gland (Table 2). Four were tested in vivo, in a rabbit conjunctival defect, by subconjunctival injection in rabbits, in a guinea pig model of form-deprivation myopia, and in a rat tarsal plate model [10,86,87,88]; six were in vitro disease or drug-screening models [9,11,89,90,91,92]. No study applied bioprinting to the iris, trabecular meshwork or ciliary body, though each was in the search strategy.

#### 3.3.1. Conjunctiva

Three included studies targeted the conjunctiva (Table 2) [9,10,86].

Dehghani et al. provided the anchor study. Using extrusion bioprinting, they fabricated a thin, transparent gelatin–elastin–hyaluronate membrane with a graded thickness mimicking native conjunctiva, thicker at the fornices and thinner near the limbus. In a rabbit conjunctival defect model, re-epithelialization time was comparable to amniotic membrane (≈21 days), but the printed membrane produced significantly less inflammation and fibrosis, twice the goblet cell density, and degraded predictably over ≈4 weeks instead of leaving the variable remnants associated with amniotic membrane [10]. The scaffold was acellular at implantation and promoted host epithelial migration; human limbal epithelial cells were used only for in vitro cytocompatibility testing.

Zhong et al. used DLP-based bioprinting to produce injectable microscale constructs of rabbit-derived conjunctival stem cells (CjSCs) in gelatin hydrogels. The micrografts retained stem cell markers including p63, exceeded 90% viability post-print, and differentiated into goblet cells in vitro; they were delivered to the bulbar conjunctival epithelium by subconjunctival injection [86].

The same group subsequently built a multicellular pterygium model, co-printing human conjunctival stem cells (hCjSCs) with vascular endothelial and immune cells [9]. The construct reproduced inflammation, angiogenesis, and epithelial–mesenchymal transition, and its transcriptomic profile corresponded to patient-derived pterygium tissue. It is the only included conjunctival study to incorporate a vascular cell population, which is relevant because the conjunctiva, unlike the cornea, is richly vascularized.

Across the three included conjunctival studies, only one delivered printed cells in vivo, and that was by subconjunctival injection of microgel constructs, not as a surgical graft [86]. No included study implanted a cell-laden, vascularized conjunctival graft.

#### 3.3.2. Sclera

One included study targeted the sclera (Table 2). Hui et al. printed a disc-shaped posterior scleral reinforcement implant from a GelMA–PEGDA bioink loaded with scleral fibroblasts, in which PEGDA provided mechanical stiffness and GelMA supported cell viability [87]. The scaffold showed controlled swelling and compressive and tensile moduli sufficient to withstand normal ocular wall stress. Implanted in a guinea pig model of form-deprivation myopia, both the plain and the fibroblast-loaded hydrogel shortened axial length over three weeks and significantly thickened the sclera by four weeks, with the fibroblast-loaded construct performing best; the encapsulated fibroblasts remained viable over seven days in vitro and secreted collagen type I [87].

#### 3.3.3. Lens and Posterior Capsule Opacification

Two included studies addressed lens-related targets, and neither attempted a complete lens (Table 2). Both addressed sub-components. Liu et al. [91] induced human mesenchymal stem cells toward a lens epithelial-like phenotype within a bioprinted matrix: a microscale sodium alginate–gelatin scaffold was loaded with MSCs and exposed to a stepwise biochemical induction regimen. Under this regimen, cells upregulated lens-specific markers, including the crystallins CRYAA and CRYAB, together with early lens developmental signaling. Viability was unaffected by encapsulation. The printed matrix alone did not induce differentiation: control constructs cultured without the inducing medium showed no sustained upregulation of crystallin genes, indicating that the sodium alginate–gelatin bioink provided a permissive three-dimensional environment, not an inductive one.

The second study modeled posterior capsule opacification (PCO), the commonest late complication of cataract surgery. Liu et al. [89] 3D-printed a transparent resin analog of the capsular bag, a hollow, thin-walled sphere with an opening and a threaded closure permitting insertion of cells or drugs and subsequent sealing, and coated its interior with a GelMA-based hydrogel to mimic the capsular basement membrane, then seeded human lens epithelial cells (LECs). A GelMA-60 5% plus 2% methacrylamide-modified ε-poly-L-lysine (PLMA) composite best supported attachment and survival with minimal cellular stress, and confocal imaging confirmed a uniform LEC monolayer with normal morphology. Eight anti-inflammatory and antiproliferative compounds were screened; rapamycin, 4-octyl itaconate, and AICAR showed potent antiproliferative effects on LECs. The platform is an in vitro drug-screening model, so no included study has printed lens tissue for implantation.

#### 3.3.4. Lacrimal System

All three included studies used bioprinting to assemble organoids or in vitro models (Table 2). Rodboon et al. labeled lacrimal acinar cells with magnetic nanoparticles and used magnetic fields to assemble spheroidal organoids without a scaffold, a technique termed magnetic 3D bioprinting (M3DB) [11]. The method produced uniform organoids at high throughput from both mouse and pig cells, which expressed the water channel aquaporin-5 (AQP5) and retained tear-secretory epithelial phenotypes in culture suitable for drug testing. Scalability and reproducibility were the stated advantages over conventional organoid culture, in which variable spheroid size and composition limit screening applications.

Ferreira et al. applied the same magnetic bioassembly platform to build paired healthy and senescent lacrimal gland models. Organoids assembled from porcine lacrimal gland cells showed epithelial hallmarks (AQP5) and measurable secretory function by lysozyme activity [90]. DNA damage-induced senescence was then produced with etoposide, giving reduced secretion, increased cell death, and senescence markers consistent with glandular aging. Transfection of the senescent organoids with a high-mobility group box 1 (HMGB1) Box A gene therapy construct protected them from the associated degeneration. Leo et al. took a materials approach, formulating a composite bioink from decellularized lacrimal gland extracellular matrix and alginate [93]. Over seven days of culture, metabolic activity was 4.8-fold at day 3 and 7.0-fold at day 7 relative to 2% alginate alone, and the Young’s modulus of the printed construct was comparable to native porcine lacrimal gland. All three lacrimal studies are in vitro models: no included study printed a lacrimal construct for implantation, and none reported sustained tear secretion beyond the culture period.

#### 3.3.5. Eyelid, Meibomian Gland and Nasolacrimal System

One included study targeted the ocular adnexa beyond the lacrimal gland (Table 2). Lee et al. bioprinted tarsal plate scaffolds from an atelocollagen–alginate bioink embedding human adipose-derived mesenchymal stem cells and implanted them into surgically created tarsal plate defects in rats. At four weeks, cell-laden scaffolds supported reconstruction of the posterior lamella, expressed meibocyte differentiation markers (SREBP-1, PPAR-γ, FADS2 and FAS), and produced no adverse events in any operated eyelid [88]. This is the only adnexal construct beyond the lacrimal gland identified by the search, and meibocyte lipid secretory function was not demonstrated. No bioprinting study targeted the nasolacrimal drainage system: published work on the canaliculi and nasolacrimal duct is confined to acellular 3D-printed stents and conduits, which are devices and fall outside the eligibility criteria of this review.

### 3.4. Cross-Study Synthesis

**Figure 4:**
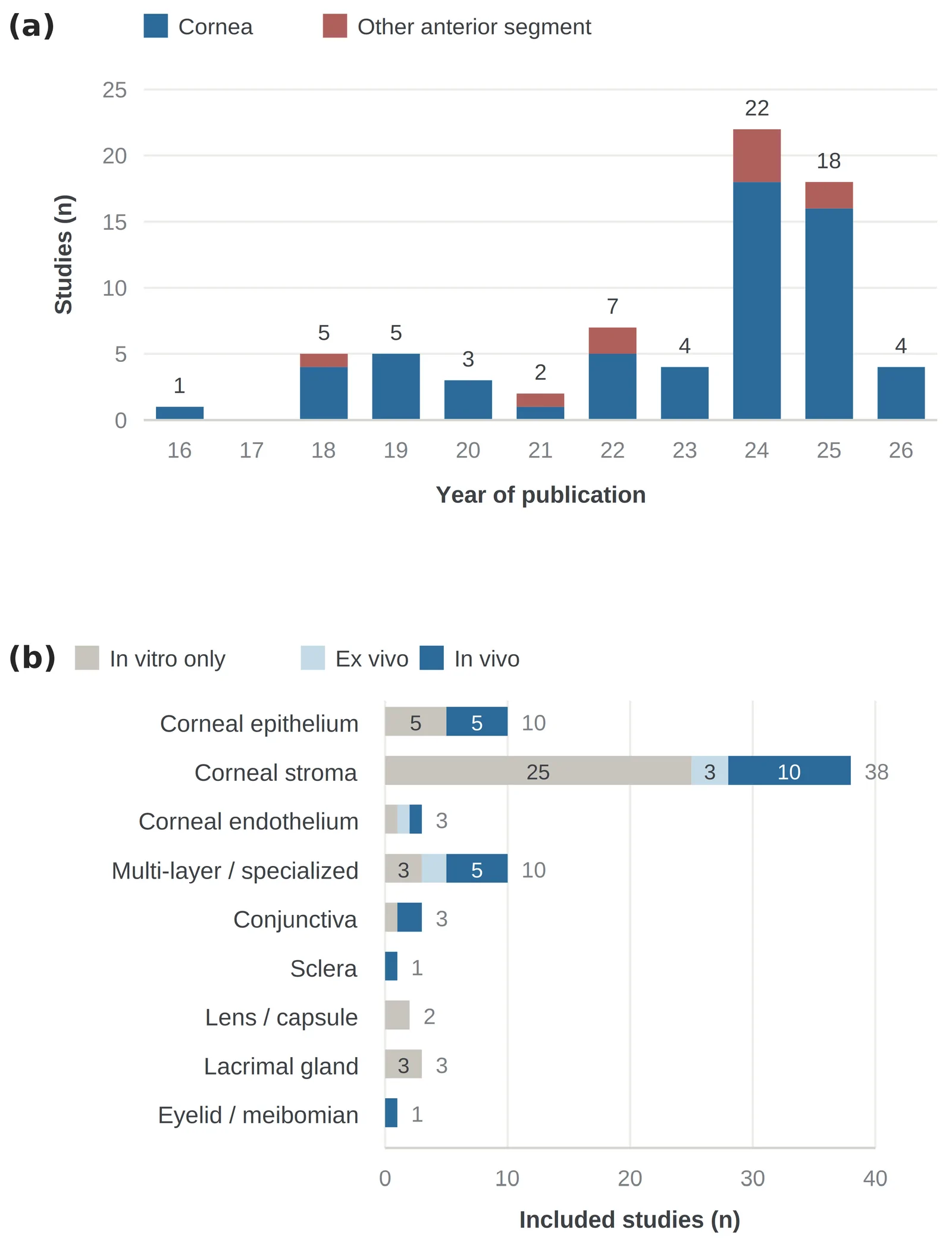

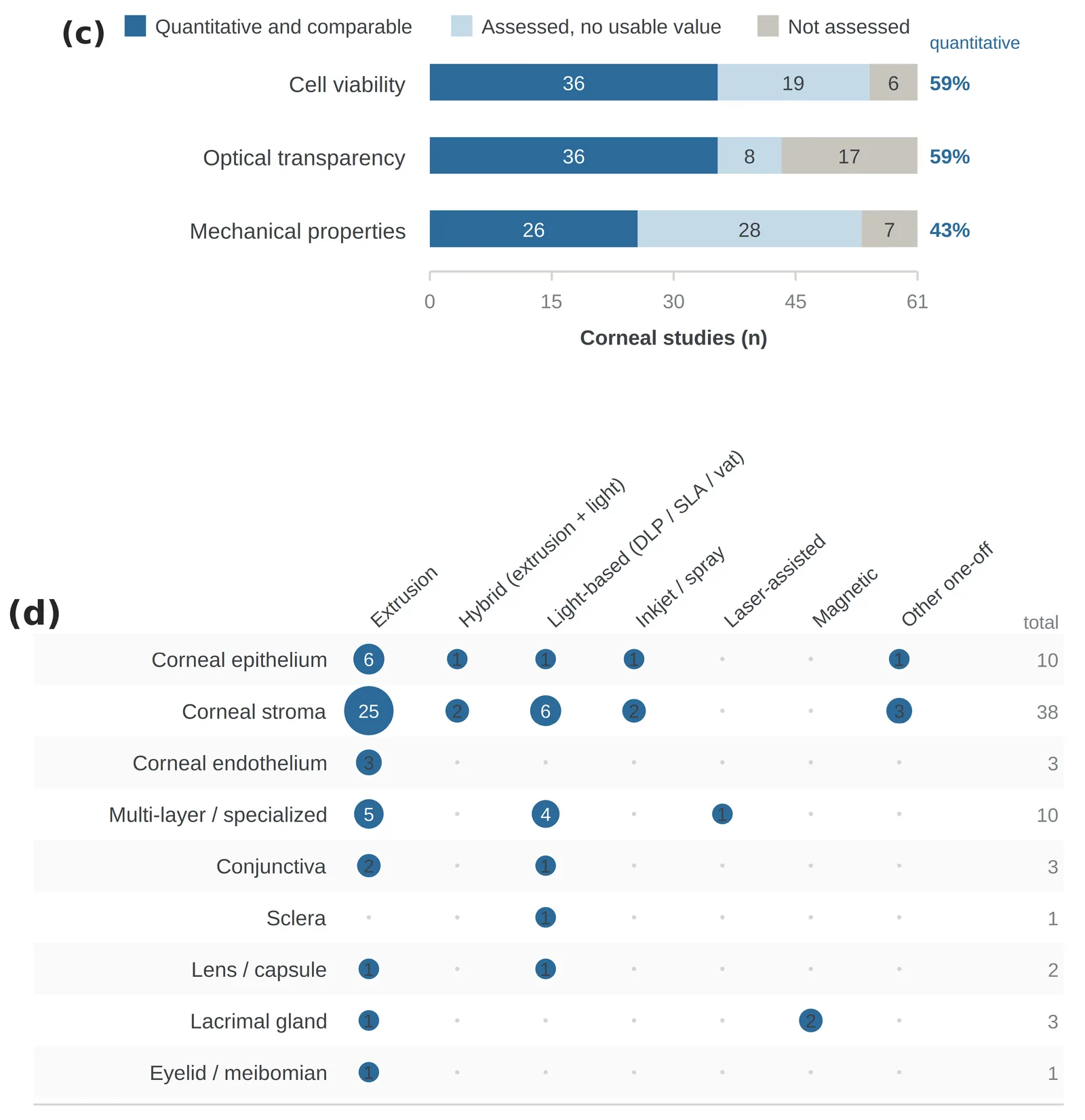
Cross-study synthesis of the 71 included studies. (**a**) Included studies by year of publication, separated into corneal targets (*n* = 61) and other anterior segment targets (*n* = 10); no eligible study was published in 2017, and 2026 is a partial year reflecting the August 2026 search cut-off. (**b**) Evidence maturity by target tissue; each study is counted once, at the highest level of evaluation it reached (in vitro only, *n* = 40; ex vivo, *n* = 6; in vivo, *n* = 25). (**c**) Completeness of outcome reporting across the 61 corneal studies; for each of cell viability, optical transparency and mechanical properties, studies are classified as quantitative and comparable (a numeric value with units and, for transparency, a stated wavelength), assessed without a usable value (qualitative statements, numeric values lacking a wavelength, or results presented only as figure curves), or not assessed. Noncorneal studies are excluded from (**c**) because optical transparency does not apply to the lacrimal gland or conjunctiva. (**d**) Bioprinting modality by target tissue; circle area is proportional to the number of studies. Each study is assigned to a single modality, and the three studies that combined extrusion with light-based patterning are shown as a separate hybrid category rather than being counted twice.

The 71 studies span 2016 to 2026, but the distribution is markedly uneven (Figure 4a). The first eight years, 2016 to 2023, contributed 27 studies, with none published in 2017; 2024 and 2025 together contributed 40 (56%), with four more in early 2026. Noncorneal work began in 2018 and peaked at four studies in 2024. This recency has two consequences: most constructs have not been independently reproduced, and follow-up is short by design, not omission.

Coverage concentrates on one layer. The corneal stroma accounts for 38 of 71 studies (54%), followed by multilayer and specialized corneal constructs (10), the epithelium (10) and the endothelium (3). The ten noncorneal studies split across conjunctiva (3), lacrimal gland (3), lens or posterior capsule (2), sclera (1) and eyelid/meibomian gland (1). None targeted the iris, trabecular meshwork or ciliary body. This distribution matches neither clinical need nor surgical volume: the endothelium, the corneal layer most often replaced surgically, is addressed by only three studies, of which one is without a viability figure.

Stratified by intended application, the corpus is dominated by the implantation and regeneration pathway. Of the 61 corneal studies, 54 pursued tissue substitution or regenerative delivery, five were in vitro disease models or screening platforms [32,38,61,63,64], and two combined regeneration with drug delivery [80,94]. Among the ten noncorneal studies, the lacrimal gland organoid work and the posterior capsule opacification platform are best read as disease modeling, while the conjunctival and scleral constructs and the lens-epithelial prototype target regeneration. The two pathways answer to different endpoints: implantation and regeneration is judged by integration, mechanical and optical function, host response and follow-up duration, whereas disease modeling and screening is judged by fidelity to human tissue, reproducibility and assay performance. Because these criteria do not overlap, a construct that is well advanced as a disease model may remain immature as an implant and the reverse.

Each study was assigned to the single modality used to pattern the construct. Extrusion-based printing dominated (44 of 71, 62%), light-based patterning by DLP, stereolithography or vat photopolymerization accounted for 14 (20%), and three studies combined the two. The remaining ten used inkjet or cell-spray (3), magnetic bioassembly (2), laser-assisted transfer (1) and four one-off modalities: aspiration-based auto-bioprinting, spiral printing, in situ deposition and a handheld biopen. The two 4D bioprinting studies are counted under extrusion, their deposition modality. This reflects instrument availability, not a demonstrated modality–tissue match: extrusion was applied even to the endothelium and epithelium, where a defined-thickness monolayer would favor the higher resolution of light- and laser-based methods.

Evidence maturity is limited (Figure 4b). Forty of 71 studies (56%) were in vitro only; six had an ex vivo component, mostly porcine organ culture; 25 (35%) included in vivo work, in rabbits (19), rats (4), one Bama piglet and one guinea pig; and one rabbit study also used a mouse model. No study used a non-human primate or reported human implantation. Where follow-up was stated, the longest was 90 days, and none exceeded three months, so neither long-term graft survival nor delayed degradation has been assessed.

The three outcome domains are reported with very different rigor, the main barrier to quantitative synthesis (Figure 4c). Among the 61 corneal studies, viability was a percentage with a named assay in 36, assessed without a usable value in 19, and not assessed in six; transparency was a percentage with a stated wavelength in 36, assessed without a usable value in eight, and not assessed in 17. Mechanics was least standardized: 26 gave a named modulus with units, 28 substituted a durability statement such as “stable”, and seven did not assess it. Compressive, tensile and storage moduli often had to be distinguished because studies used the single word “modulus” for different quantities.

Even where an outcome was quantified, measurement conditions differ too widely to pool. Viability was assessed from immediately postprinting to day 21 by live/dead, MTT, CCK-8 and alamarBlue on constructs of differing thickness and cell density; transparency at single wavelengths (550, 600, 700 nm) and across several ranges, hydrated or dehydrated, with or without cells. No two studies share the same assay, timepoint and wavelength across all three domains. Meta-analysis was not attempted, and the synthesis above is narrative.

## 4. Discussion

### 4.1. Implantation and Regeneration: Current Progress

The distribution of this literature is itself informative: research effort has tracked technical tractability more closely than clinical need. Coverage and maturity are distinct measures. Coverage counts how many studies target a tissue; maturity reflects how far its constructs have been validated against the intended application. A cross-tissue comparison of clinical readiness is provided in Table 3. The cornea has dominated, and the scope is only now widening to other anterior segment structures, where implants [10,87] and laboratory models are represented in equal number. Across these tissues, the distinctive contribution of bioprinting is anatomically specific, patient-matched constructs that address structural causes of disease directly, which off-the-shelf materials and systemic drugs cannot. In the regeneration pathway, printed constructs act as cell-delivery vehicles as well as tissue substitutes. One example is a limbal stem cell niche printed with defined stiffness and growth factors, offering a route to treat limbal stem cell deficiency [8,29].

The lens and capsule studies are preliminary but mark first steps toward a bioengineered lens substitute. Differentiating stem cells to a lens epithelial phenotype on a printed matrix is a prerequisite for autologous lens regeneration after cataract surgery [91]. The bioprinted PCO model [89] gives an in vitro platform to test anti-scarring drugs [92,95] in a geometry that reproduces the capsular bag, which could help identify agents applied at surgery to prevent posterior capsule opacification, a complication with a reported five-year incidence of about 12 to 34% that varies with intraocular lens material and edge design [96,97,98]. The lacrimal gland organoids show a second use: physiologically relevant glandular models that can be driven into disease states such as aging and used to test therapies, including the gene therapy that reversed organoid senescence [11,90,99]. These sit between 2D culture and animal work, which matters for dry eye disease, where lacrimal gland immunology is hard to reproduce in animals.

The reporting heterogeneity has a methodological corollary: blinding and randomization were inconsistently reported in the animal studies, and in vitro work often lacked appropriate controls, such as a bioprinted versus conventionally cultured comparison. Standardized outcome measures, longer in vivo validation, and transparent reporting are needed to reduce bias.

The corneal endothelium raises a question specific to this layer: whether printing is necessary at all. Because the endothelium is a single 5–10 μm monolayer [100,101], cultured cells can be injected into the anterior chamber and allowed to attach [102], an approach that has progressed from first-in-human study to an approved and reimbursed therapy in Japan [103]. Injected cells have sustained endothelial function in treated eyes for five years [104]. The claimed advantage of bioprinting is delivery of a pre-arranged monolayer at uniform density on a supporting matrix that can also carry topographic cues for cell orientation, and the in vivo result of Kim et al. is consistent with this, in that a printed monolayer adhered and functioned immediately [77]. Two further differences follow from the delivery route. Injection depends on cells settling onto a receptive posterior surface, so eyes with a damaged Descemet’s membrane or a retrocorneal fibrous membrane are poor candidates [103], whereas a carrier-borne monolayer does not require that surface. A printed construct can also be characterized for confluence, density and pump-marker expression before implantation, which converts attachment efficiency from an in vivo variable into a release specification. The two approaches have not been compared directly, however, and this remains the central unresolved question for the endothelium. The stakes are considerable, since roughly one third of corneal transplants worldwide are endothelial keratoplasty procedures, making the endothelium the most frequently replaced single corneal layer [1,36,105]. Whether printed endothelial cells retain density and pump function over months to years, and attach without fibrocellular overgrowth, is untested. That question is now being asked clinically. In October 2025, a cell-based bioprinted corneal endothelial implant (PB-001) was transplanted into the first patient of a Phase 1 single-arm trial in corneal edema from endothelial dysfunction [106]. The endothelium reached first-in-human ahead of every other anterior segment tissue. This is a company report of a trial in progress and not peer-reviewed primary research, and it did not meet the inclusion criteria applied here. That trial is single-arm, so it cannot resolve the comparison with injection. No outcome advantage of printing has been demonstrated, and the question would be settled by a head-to-head trial in eyes with a compromised posterior surface, where the two routes are not interchangeable.

Taken across the corneal layers, the field has moved from single-layer sheets to multilayered constructs that demonstrate optical function and short-term in vivo integration. The remaining barriers are consistent across studies: adhesion between printed layers and to the host corneal rim, long-term endothelial function so that the graft stays clear, and immune rejection. Corneal immune privilege mitigates the last of these, but any cell-laden implant may provoke inflammation unless autologous or universal donor cells are used. Set against this, print resolution, bioink biocompatibility, and optical performance are now largely tractable, and several bioprinted grafts have restored corneal clarity in animal models. On present evidence, the cornea is the anterior segment tissue closest to a bioprinted clinical product, and the first bioprinted endothelial construct has now entered a first-in-human trial.

### 4.2. Bioprinting and Additive Refractive Correction: A Paradigm Shift?

One application follows directly from the lenticule studies identified here: bioprinted corneal lenticules for refractive correction [19]. Keratorefractive surgery, including laser-assisted in situ keratomileusis (LASIK), photorefractive keratectomy (PRK) and small-incision lenticule extraction (SMILE), corrects vision by ablating or removing stromal tissue [107,108]. This is subtractive and irreversible. It leaves little margin for retreatment, weakens the cornea, and can trigger ectasia when deeper ablations are needed [109,110]. Outcomes also degrade at the extremes of refractive range, with reduced predictability in high myopia (≥−10 D) and poor long-term stability in hyperopic correction [111,112,113].

Additive correction offers a different route: adding a shaped optical element instead of removing tissue, which could preserve or strengthen the cornea. Keratophakia, implanting a lenticular graft, dates back decades, but only recently have tools allowed lenticules to be made with sufficient precision [114]. Lenticule intrastromal keratoplasty (LIKE) using lenticules extracted from SMILE has treated hyperopia and keratoconus with encouraging results, and cryopreserved SMILE-derived lenticules implanted in post-LASIK ectasia improved acuity and added about 40 μm of corneal thickness over one year [111,115,116,117].

**Figure 5:**
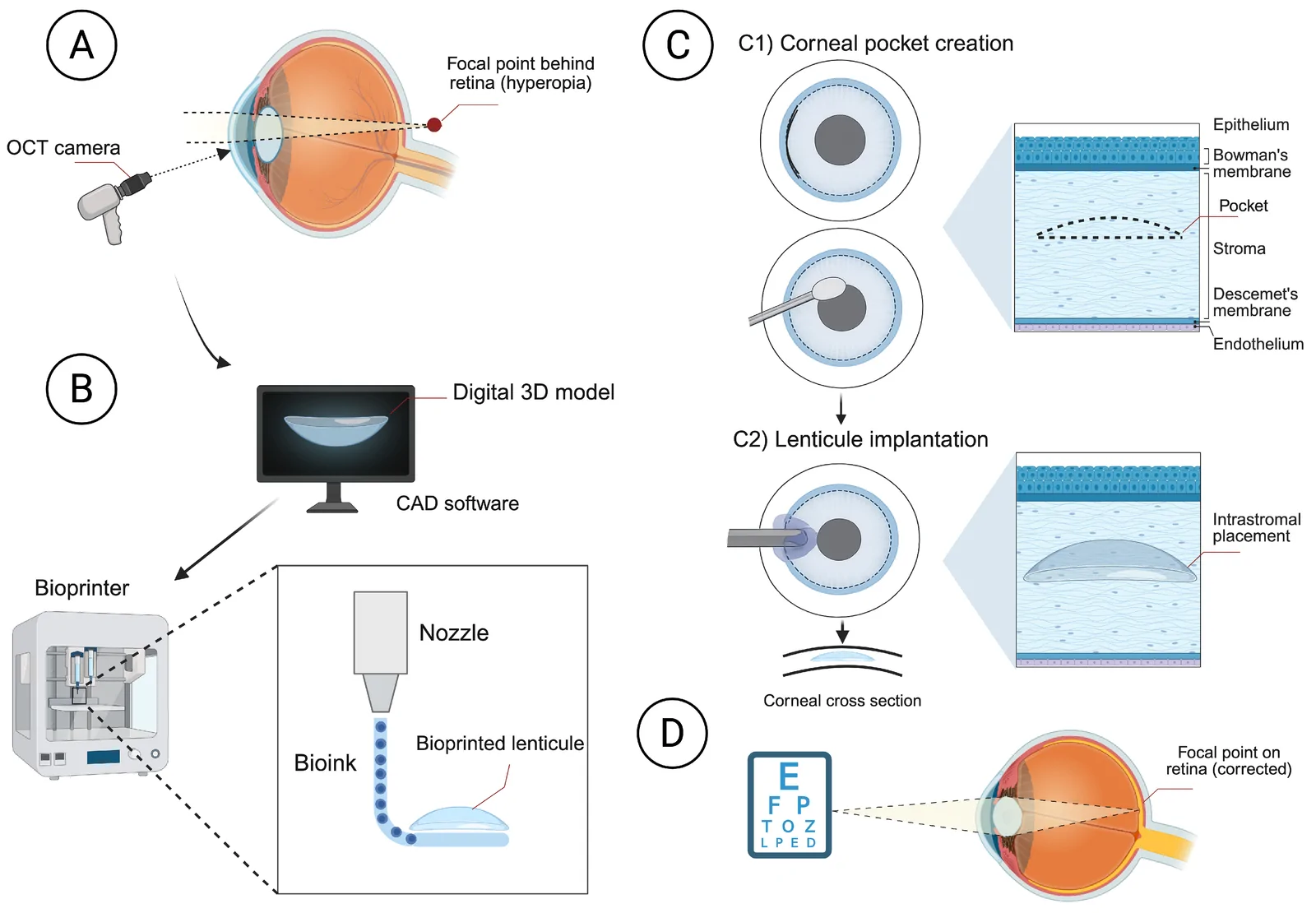
Schematic of a proposed workflow for bioprinted intrastromal lenticule implantation, illustrated for hyperopic correction. The schematic shows how patient-specific imaging, digital design and bioprinting could together produce a customized biconvex lenticule without donor tissue. Hyperopia is the illustrative indication because central steepening is the geometry least well served by ablation; the workflow applies to keratoconus and post-refractive ectasia. (**A**) Hyperopic eye evaluation: light focuses behind the retina; optical coherence tomography (OCT) measures parameters for lenticule design. (**B**) Digital modeling and bioprinting: computer-aided design (CAD) software generates a 3D model, exported to a bioprinter for layer-by-layer fabrication using bioink (e.g., GelMA with cells) to produce the lenticule. (**C1**) Corneal pocket creation: a precise intrastromal pocket is formed via femtosecond laser, with corneal layers labeled (epithelium, Bowman’s membrane, stroma, Descemet’s membrane, endothelium). (**C2**) Lenticule implantation: the bioprinted lenticule is inserted into the stromal pocket. (**D**) Post-implantation outcome: the lenticule integrates, redirecting light to focus on the retina for corrected vision, illustrated by a clear Snellen chart. Created with BioRender.com.

Bioprinting extends this idea to donor-free, patient-specific lenticules. Jia et al. gave proof of principle: a DLP-printed poly(NAGA)-GelMA lenticule transmitting at or above 85% at 630 nm, with a refractive index of 1.37 and 46 D of power, made to defined curvature and diopter strength [19]. Fabricated in vitro under controlled conditions, such lenticules could be more predictable than ablation and, in principle, removable or exchangeable if the target is missed. They may also preserve biomechanics: because central tissue removal reduces corneal stiffness, an integrated lenticule could maintain or augment strength and widen eligibility to thinner corneas currently excluded from surgery [73]. This biomechanical claim is untested, as no bioprinted lenticule has been implanted in a human eye. Realization requires a biocompatible, transparent, dimensionally stable lenticule, and several bioinks now cure into high-strength transparent stroma substitutes [19,64,65,68,118,119]. Cellularized lenticules may integrate better but complicate regulatory approval, whereas an acellular lenticule resembles a device yet must still integrate without remodeling. This defines a concrete research direction (Figure 5): on-demand lenticules of prescribed curvature, implanted additively and potentially reversible, whose durability over years will decide clinical viability.

### 4.3. In Vitro Disease Modeling and Drug Screening

In the in vitro modeling pathway, bioprinting builds 3D ocular disease models that reproduce the tissue microenvironment more faithfully than 2D culture. The pterygium model and the lacrimal gland organoids show this, and comparable approaches could yield models of ocular surface tumors, conjunctival scarring (e.g., Stevens–Johnson syndrome) or glaucoma angle tissues, for instance a printed trabecular meshwork for drug testing [11,90,100,120]. A printed cornea with epithelial, stromal and endothelial components could be infected ex vivo to study host–pathogen interactions and test antimicrobials, replacing some animal models [6]. The included models establish tissue-like phenotype and measurable responses to injury or treatment. Demonstrating predictive validity for clinical response would require benchmarking against human tissue and independent reproduction.

### 4.4. Future Directions: 4D Bioprinting, AI, and Unaddressed Tissues

In 4D bioprinting, constructs change shape or properties over time in response to stimuli [27,34,56,121,122]. A 4D-printed corneal patch could be flat for insertion then curve to match the cornea at body temperature, improving fit; shape-memory and stimuli-responsive bioinks that morph after fabrication are being developed [56,123].

Machine learning (ML) and artificial intelligence (AI) are increasingly used for parameter tuning and real-time quality control [124]. ML has predicted cell viability and print fidelity from input parameters, cutting the trial-and-error of bioink development [124,125]. In ophthalmology, AI could personalize implant design from patient imaging, for instance tailoring a patch graft to a defect or setting lenticule thickness for a target refraction [126,127].

Two concepts remain earlier-stage. Bioprinting of commensal bacteria has been proposed to correct ocular-surface dysbiosis in dry eye and keratitis through structured microbial delivery [128]. Bioprinting with optogenetic tools allows light control of cellular activity [129]; not yet applied to the eye, it could enable light-responsive iris constructs that mimic pupillary dynamics.

Two anterior segment tissues remain effectively unaddressed and warrant comment. For the sclera, bioprinted constructs could in principle extend beyond myopia control to repair of staphylomas, patching of defects after tumor excision, trauma, or inflammatory disease, and localized drug delivery in conditions such as scleritis. Reproducing the fiber alignment that underlies scleral tensile strength is the principal fabrication challenge; printing in a circumferential pattern is one possible solution. That the sclera is relatively acellular, non-transparent, and clinically substitutable with donor or synthetic patches likely explains why bioengineering it has been less urgent than the cornea or the lacrimal gland. For the iris, adjacent work is suggestive even in the absence of any bioprinting study: micropatterned surfaces have been shown to guide concentric ring formation in myoblast cultures, a proof of concept for sphincter-like constructs [130]. Translation would require both pigment-producing cell sources and functional contractility, and would likely depend on the light-responsive strategies described above.

### 4.5. Regulatory and Translational Challenges

The translation of 3D bioprinting for anterior segment ophthalmology into clinical applications presents a unique set of regulatory and manufacturing challenges [131]. Unlike traditional biologics or implants, bioprinted ocular tissues, such as corneal lenticules, conjunctival scaffolds, or limbal stem cell constructs, blur the lines between medical devices, biologics, and tissue-engineered products. Precedent now exists for cell-based ocular products: cultured endothelial cell therapy is approved and reimbursed in Japan [103]. No printed cell-laden construct has been approved in any jurisdiction, so the remaining uncertainty is specific to printed constructs. It affects developers working across a range of anterior segment indications, from transparent corneal implants to conjunctival scaffolds, where product classification directly impacts trial design, manufacturing requirements, and approval pathways. A core regulatory requirement is that bioprinted ocular implants must demonstrate efficacy that is at least comparable, if not superior, to conventional corneal treatments in relevant clinical endpoints such as transparency, integration, and visual acuity restoration [132].

In the European Union, such products typically fall under the scope of Advanced Therapy Medicinal Products (ATMPs) as defined by Regulation (EC) No. 1394/2007 [133]. These therapies are subject to centralized evaluation by the European Medicines Agency (EMA) and the Committee for Advanced Therapies (CAT), which assess quality, safety, and efficacy based on risk-adapted guidelines [134]. In the U.S., the Food and Drug Administration (FDA) regulates bioprinted constructs under various sections of Title 21 of the Code of Federal Regulations (CFR), with oversight varying depending on cell content, manipulation, and intended use. Human cells and tissues that are minimally manipulated and intended for homologous use are regulated under Section 361 of the Public Health Service Act, while products failing these criteria, including bioprinted constructs incorporating expanded cells, are regulated as biological products under Section 351 of the Public Health Services Act and must meet current Good Manufacturing Practice (cGMP) standards [134,135].

Manufacturing reproducibility and quality control raise requirements for bioprinted ocular implants that go beyond those for conventional devices. Batch release for these constructs depends on demonstrating cell identity, viability and potency, not on dimensional conformity alone [133,134,135]. The FDA guidance on additive-manufactured devices states explicitly that products incorporating biological, cellular or tissue-based components may require additional considerations or a different regulatory pathway, since process parameters qualified once for a polymer device must be re-qualified against a biological output [136]. Ocular implants add three product-specific specifications to that burden: optical transmittance across the visible spectrum, mechanical properties sufficient for suturing or sutureless apposition, and sterility achieved without degrading either. The last is not hypothetical, since terminal sterilization and thermal gelation measurably reduced transparency in a bioprinted stromal construct [76]. Comparable scalability, sterilization and batch-variability constraints have been documented for polysaccharide-based ocular formulations, although those systems are acellular and their controls do not transfer directly to living constructs [137]. GMP-compliant production must extend across bioink preparation, printer calibration, aseptic handling, and environmental validation.

Industrial players are beginning to address these challenges. Precise Bio manufactures GMP-compliant bioprinted corneal grafts at a dedicated facility and has since progressed from animal models to a first-in-human Phase 1 trial. The manufacturing platform and preclinical data have been presented in abstract form, while the clinical outcomes remain company-reported; together these developments underscore the importance of industry–academic partnerships to scale up production, standardize biofabrication, and de-risk regulatory approval [106].

### 4.6. Ethical, Economic, and Accessibility Considerations

Beyond regulatory classification, the choice between autologous and allogeneic constructs poses further translational dilemmas. While autologous cell-laden implants offer immune compatibility, they require individualized processing, biopsy expansion, and longer lead times, factors that limit scalability. Universal donor cell lines or acellular, immune-evasive bioinks may streamline access but introduce new regulatory burdens and ethical considerations [138].

Equity and cost are also pivotal. With over 12 million people worldwide affected by corneal blindness, any viable bioprinted solution must be both scalable and affordable [1]. Reports of the Phase 1 manufacturing run describe a single donor cornea being expanded and printed into roughly 300 implants [139], which indicates the order of magnitude of the supply change at stake. Anterior segment applications like refractive lenticules or acellular stromal scaffolds may offer an entry point due to their mechanical simplicity, shorter implantation times, and relatively low cell requirements compared to posterior segment constructs. These attributes position anterior segment bioprinting as an ideal starting domain for clinical trials and early regulatory engagement. As the field advances, standardized reporting, precompetitive data sharing, and international regulatory harmonization will be critical.

### 4.7. Toward a Minimum Reporting Set for Ocular Bioprinting

The reporting heterogeneity documented in this review is a field-level problem, and a fixable one. Across the 71 studies, transparency was reported at six wavelengths and six ranges, viability from immediately postprinting to day 21 across four assays, and mechanics with explicit units in fewer than half of the corneal studies (Figure 3 and Figure 4c). No two studies shared the same combination of assay, timepoint and measurement condition, so quantitative synthesis was not possible. Transparency is not even defined consistently: most studies measured spectrophotometric transmittance, while others graded corneal opacity on an ordinal clinical scale from slit-lamp photographs, an outcome that cannot be placed on the same axis as a percentage. A short, discipline-specific reporting minimum would remove most of this barrier. Building on the ARRIVE guidelines for animal research [140], we propose the set in Table 4, limited to the measurements that decide whether one construct can be compared with another. We intend it as a starting point for the community to refine, not a fixed standard; even partial adoption would make future studies comparable and, in time, poolable.

### 4.8. Stromal Cell Phenotype, Fibrosis, and Long-Term Haze

Every stromal construct in this review depends on a cell that changes identity when disturbed. Quiescent keratocytes are dendritic, largely nonproliferative, and express keratocan, lumican and crystallin-family proteins that contribute directly to stromal transparency. Isolation, serum exposure and expansion convert them to fibroblasts, and TGF-β signaling in a stiff or provisional matrix drives further conversion to α-smooth muscle actin (α-SMA)-positive myofibroblasts that deposit disorganized collagen and fibronectin. That end state is the cellular substrate of clinical haze [141]. Bioprinting does not simply place keratocytes: it exposes them in sequence to nozzle shear, photoinitiator radicals, altered substrate stiffness and a non-native matrix, each a documented trigger of the same transition.

Reporting against this framework is uneven across the thirty-eight stromal studies. Phenotype retention was most often evidenced by keratocan and lumican expression, as in the first printed stromal equivalent [31] and in corneal dECM inks [49,54,55], and matrix-specific inks consistently outperformed synthetic hydrogels on these markers, consistent with keratocyte identity depending on biochemical cues and not on mechanics alone. Only a minority reported the complementary measurement, α-SMA, which is what would establish the absence of myofibroblast conversion. Choi et al. observed downregulation of fibrotic markers after four weeks in printed human stromal cells [32]. The asymmetry matters: keratocan expression in a population does not exclude an activated subpopulation, and a construct can be transparent at implantation while carrying cells already committed to a fibrotic phenotype.

ECM organization is where printing has most to contribute and where the evidence is strongest. Shear-aligned fibrils reproduce lamellar orientation and improve both strength and clarity, since ordered fibrils scatter less light [49,55], and dome-shaped implants integrated with less fibrosis than flat constructs of identical composition [33]. Substrate stiffness cuts the other way. Matching the modulus of donor cornea requires crosslinking that moves the construct toward the stiffness range in which myofibroblast conversion is favored, so the mechanical target set by suturing and the biological target set by phenotype are in partial conflict. No included study addressed this trade-off explicitly, and none reported fibril organization and α-SMA status in the same construct.

Long-term haze is essentially unaddressed. The longest in vivo follow-up in this review was 90 days [74], whereas haze after stromal injury evolves over months and may either resolve or consolidate well beyond that window. No included study tracked α-SMA, collagen III or objective backscatter over such an interval, and none used a model of pre-existing scarring, which is the clinical population a stromal substitute would actually treat. Assessing haze properly requires endpoints the field does not yet report as standard: myofibroblast markers at the construct–host interface, densitometry by confocal microscopy or Scheimpflug imaging in place of transmittance measured on the explanted construct, and follow-up matched to the remodeling timeline. These are incorporated into the minimum reporting set proposed above.

### 4.9. Limitations of This Review

Several limitations apply. The search covered only PubMed and Scopus and was restricted to English, so eligible studies in other databases or languages may have been missed. Studies were appraised for reproducibility, translational relevance and reporting completeness, not with a validated risk-of-bias instrument, and the reporting heterogeneity precluded meta-analysis, so no pooled estimates are given. The evidence base is also young and short-term: most studies date from 2024 onward and the longest in vivo follow-up was 90 days, so durability cannot yet be judged. Finally, the synthesis relies on author-reported outcomes without access to raw data.

## 5. Conclusions

Within a decade, anterior segment bioprinting has moved from proof of concept to a first-in-human trial, but it has done so unevenly. Cell-laden constructs for the cornea, conjunctiva, sclera and eyelid have been printed and implanted in animal models, whereas lens and lacrimal gland work remains confined to in vitro models. This heterogeneity follows tissue biology, not research effort: a tissue is more advanced when its geometry is simple, its cell source is defined, it needs no vascular supply, and its functional endpoint is measurable. The corneal stroma and the ocular surface epithelia meet these conditions and are closest to the clinic; the endothelium is favorable in geometry yet thin in evidence; the lens is furthest behind, and lacrimal gland work is directed at laboratory models rather than implants. The distinct promise of bioprinting is not only to replace tissue but to improve on it, for example a graft that resists infection or a removable lenticule that corrects refraction without ablation. Three barriers remain common to every tissue: durable function beyond the current 90-day ceiling, a defined regulatory path, and a scalable, immune-compatible cell source. Progress now depends less on printers and bioinks, which are largely adequate, and more on closing these gaps. Adopting a common minimum reporting set is the most immediate of these steps and the one most fully within the field’s control. The next decade will show whether early constructs hold optical and functional performance over years and whether anterior segment bioprinting reaches routine clinical use.

## Figures and Tables

**Table 1:** Comparative summary of representative corneal bioprinting studies, grouped by subsection of Section 3.2. Studies were selected to represent distinct fabrication strategies or the strongest functional validation within each subsection; the complete inventory of included studies is provided in Table S2. Outcomes follow the review’s reporting convention: viability as a percentage with assay and timepoint, transparency as a percentage with the measurement wavelength, and mechanics as the named modulus with its value. Transparency values are not directly comparable across studies because the measurement wavelength differs.

Study [Ref]	Modality (ASTM Family)	Bioink (Crosslink)	Model (Follow-Up)	Viability	Transparency (λ)	Mechanics
**Section 3.2.1**						
Wang et al. 2024a [27]	Extrusion (4D)	Chitosan/CMC (thermosensitive)	In vivo, diabetic rabbit	>90% (post-print)	N/R	Porous scaffold
Kauppila et al. 2025 [61]	Extrusion	HA-based + PA-gel scaffold	In vitro	>90% (1 wk)	N/R	Native-like
**Section 3.2.2**						
Isaacson et al. 2018 [31]	Extrusion (FRESH)	Alginate/collagen-MA (CaCl_2_ + LAP)	In vitro	92% (d1); 83% (d7)	N/R	~600 µm; stable 7 d
Kim et al. 2018a, 2019a [54,55]	Extrusion	Cornea-derived dECM (thermal)	In vivo (mouse 8 wk; rabbit 4 wk)	N/R	>75% (300–700 nm)	Stable 28 d
Kim et al. 2019b [49]	Extrusion	Collagen + MSCs (shear-aligned)	In vivo, rabbit 4 wk (*n* = 10)	N/R	Measured 300–700 nm	Aligned fibrils
Basoz et al. 2025 [74]	Extrusion	Keratocyte-laden GelMA (photo)	In vivo, rabbit 90 d	Qual. (live/dead, d1–21)	~60% (400 nm); ~70% (700 nm)	Compressive ~ 281 kPa
**Section 3.2.3**						
Kim et al. 2018b [77]	Extrusion	RNase 5-HCECs on amniotic membrane	In vivo, rabbit	N/R	Clinical clarity (qual.)	N/R
Grönroos et al. 2024 [37]	Extrusion	HA (hydrazone crosslink)	Ex vivo (rat/porcine/human DM)	~92.5% (d7)	N/R	26% mass at d10
Overmass et al. 2024 [78]	Extrusion	Collagen type IV	In vitro	Qual. (confluent)	97.8 ± 0.5% (400–700 nm)	Tensile 89.5 kPa
**Section 3.2.4**						
Sorkio et al. 2018 [28]	Jetting (laser-assisted)	HA/collagen (LSCs + ASCs)	Ex vivo, porcine organ culture	Qual. (live/dead, d 1–7)	N/R	Stable integration
He et al. 2022 [30]	Vat (DLP)	PEGDA-GelMA	In vivo, rabbit ALK	~90% (d 7)	90.7% (600 nm)	Compressive ~ 100 kPa
**Section 3.2.5**						
Jia et al. 2023 [19]	Vat (DLP)	GelMA refractive lenticule (photo)	In vivo, rabbit 4 wk	>90%	>85% (630 nm)	~ native cornea
Ghosh et al. 2025 [85]	Extrusion (multi-material)	Silk fibroin/decell. matrix (dopamine)	In vitro; ex vivo	>85–90% (d14)	90% (700 nm)	Adhesion ~ 85 kPa

Abbreviations: ALK, anterior lamellar keratoplasty; ASCs, adipose-derived stem cells; ASTM, ASTM International; CaCl_2_, calcium chloride; CMC, carboxymethyl cellulose; dECM, decellularized extracellular matrix; DLP, digital light processing; DM, Descemet’s membrane; FRESH, freeform reversible embedding of suspended hydrogels; GelMA, gelatin methacryloyl; HA, hyaluronic acid; HCECs, human corneal endothelial cells; LAP, lithium phenyl-2,4,6-trimethylbenzoylphosphinate; LSCs, limbal epithelial stem cells; MA, methacrylated; MSCs, mesenchymal stem cells; N/R, not reported; PA, polyacrylamide; PEGDA, poly(ethylene glycol) diacrylate; Qual., viability confirmed qualitatively (e.g., live/dead staining) without a reported percentage; RNase, ribonuclease.

**Table 2:** Summary of included studies of 3D bioprinting for noncorneal anterior segment tissues. Summary of all included peer-reviewed studies (2018–2025) on 3D bioprinting applied to anterior segment tissues beyond the cornea, including conjunctiva, sclera, lens, lacrimal gland, and eyelid/meibomian gland. For each study, the table reports the publication year, targeted tissue, application, cell source, bioink composition (with crosslinking method where applicable), bioprinting technique, experimental model (in vitro or in vivo, including animal species and follow-up duration where reported), and notable biological or functional outcomes. All studies meeting the inclusion criteria outlined in Materials and Methods are listed.

Reference (Year)	Tissue Targeted	Application	Cell Source	Bioink	Bioprinting Technique	Model (In Vitro/In Vivo)	Notable Outcomes
Dehghani et al. (2018) [10]	Ocular surface/conjunctiva	Conjunctival defect reconstruction as alternative to amniotic membrane	Acellular at implantation; human limbal epithelial cells used for in vitro cytocompatibility	Gelatin/elastin/sodium hyaluronate (UV photocrosslink with Irgacure 2959)	Extrusion-based	In vitro; in vivo (rabbit, ~4 wk)	More predictable degradation than amniotic membrane; reduced inflammation and scar formation in rabbit conjunctival defects
Zhong et al. (2021b) [86]	Conjunctiva	Injectable conjunctival stem cell micro-constructs for subconjunctival ocular injection	Conjunctival stem cells (rabbit)	Alginate/gelatin/fibrinogen (CaCl_2_ ionic crosslink)	DLP	In vitro; in vivo (rabbit, subconjunctival injection; follow-up N/R)	>90% viability post-print; maintained stemness (p 63, ABCG2); integrated post-injection; reduced inflammation in vivo
Zhong et al. (2022) [9]	Conjunctiva	Multicellular model recapitulating pterygium microenvironment	Conjunctival epithelial cells, fibroblasts, immune cells (human)	Gelatin/alginate/collagen (CaCl_2_ ionic crosslink)	Extrusion-based	In vitro	Qualitative (post-print viability); fibrotic/inflammatory markers (α-SMA, IL-6); recapitulated pterygium pathology; N/R transparency/mechanics
Rodboon et al. (2022b) [11]	Lacrimal gland	High-throughput lacrimal gland organoid platforms for drug discovery in dry eye disease	Lacrimal gland epithelial cells (mouse, porcine)	Magnetic nanoparticles (no hydrogel; magnetic assembly)	Magnetic bioassembly	In vitro	Qualitative (post-print viability and proliferation); secretory function (lysozyme); maintained acinar morphology and AQP5 expression
Ferreira et al. (2024) [90]	Lacrimal gland	Unveiling senescence-associated ocular pathogenesis via lacrimal gland organoid platform and HMGB1-Box A gene therapy	Lacrimal gland epithelial cells (porcine)	Magnetic nanoparticles (no hydrogel; magnetic assembly)	Magnetic bioassembly	In vitro	Qualitative (post-assembly viability); senescence-associated secretory phenotype prevented by HMGB1-Box A; multi-omic protection
Liu et al. (2025) [91]	Lens	Differentiation of mesenchymal stem cells towards lens epithelial stem cells based on a 3D bioprinted matrix	Mesenchymal stem cells (human)	Sodium alginate–gelatin (CaCl_2_ ionic crosslink)	Extrusion-based	In vitro	Qualitative (post-print viability); upregulated LSC markers (CRYAB, CRYAA)
Liu et al. (2024) [89]	Lens (posterior capsule)	Fabrication of a 3D bioprinting model for posterior capsule opacification	Lens epithelial cells (human)	GelMA/PLMA hydrogel (UV photocrosslink)	Resin 3D printing (vat photopolymerization) + hydrogel spin-coating	In vitro	Qualitative (post-print viability); fibrotic tissue (α-SMA expression); model mimics PCO pathology
Hui et al. (2024) [87]	Sclera	3D-printed fibroblast-loaded hydrogel for scleral remodeling to prevent myopia progression	Scleral fibroblasts (human)	GelMA–PEGDA (DLP photocrosslink)	DLP	In vitro; in vivo (guinea pig, form-deprivation myopia; 4 wk)	Qualitative (post-print viability); reduced axial elongation in myopic model
Leo et al. (2025) [93]	Lacrimal gland	In vitro lacrimal gland model for aqueous-deficient dry eye	Lacrimal gland epithelial cells and mesenchymal stromal cells (porcine); HUVECs	Porcine LG dECM + alginate (LGnate); CaCl_2_ ionic crosslink	Extrusion	In vitro (7 days)	Metabolic activity 4.8 × (day 3) and 7.0 × (day 7) vs. 2% alginate; Young’s modulus comparable to native porcine LG
Lee et al. (2024) [88]	Eyelid/Meibomian gland (tarsal plate)	Tarsal plate reconstruction	Adipose-derived MSCs (human)	Atelocollagen/alginate (N/R)	Extrusion-based	In vivo (rat, tarsal plate defect; 4 wk)	hADSC-laden scaffolds supported tarsal plate reconstruction; meibocyte differentiation markers (SREBP-1, PPAR-γ, FADS2, FAS) expressed; no adverse events at 4 weeks

Abbreviations: ABCG2, ATP-binding cassette sub-family G member 2; AQP5, aquaporin-5; CaCl_2_, calcium chloride; CRYAA, crystallin alpha A; CRYAB, crystallin alpha B; dECM, decellularized extracellular matrix; DLP, digital light processing; FADS2, fatty acid desaturase 2; FAS, fatty acid synthase; GelMA, gelatin methacryloyl; hADSC, human adipose-derived stem cell; HMGB1-Box A, high-mobility group box 1 protein Box A domain; HUVECs, human umbilical vein endothelial cells; IL-6, interleukin-6; LG, lacrimal gland; LGnate, lacrimal gland dECM–alginate composite bioink; LSC, limbal epithelial stem cell; MSCs, mesenchymal stem cells; N/R, not reported; p63, tumor protein p63; PCO, posterior capsule opacification; PEGDA, poly(ethylene glycol) diacrylate; PLMA, methacrylamide-modified ε-poly-L-lysine; PPAR-γ, peroxisome proliferator-activated receptor gamma; SREBP-1, sterol regulatory element-binding protein 1; UV, ultraviolet; α-SMA, alpha-smooth muscle actin.

**Table 3:** Translational readiness of bioprinted constructs in the implantation and regeneration pathway (anterior segment, as of 2026). Summary of clinical needs, examples of reported bioprinted constructs (illustrative from 2018–2026 literature), translational barriers, preclinical testing, and estimated readiness levels for anterior segment ocular disorders. Readiness is scored on five domains, each 0–3, giving a total of 0–15. D1, quantity and quality of preclinical evidence—0, none; 1, one to two studies; 2, three to nine; 3, ten or more—with one point deducted if fewer than half the studies for that tissue report a quantified primary outcome with stated units and measurement conditions. D2, highest level of evaluation: 0, in vitro only; 1, ex vivo; 2, in vivo in a single study; 3, in vivo in multiple studies. D3, duration of in vivo validation: 0, none; 1, up to two weeks; 2, over two to four weeks; 3, over four weeks. D4, functional outcome: 0, viability or histology only; 1, structural integration; 2, tissue-appropriate function demonstrated; 3, function sustained to the end of follow-up and quantified. D5, clinical relevance of the model: 0, in vitro or organoid; 1, rodent; 2, large-eye species; 3, disease-matched model in a large-eye species. Totals map to tiers: Very Low, 0–3; Low, 4–6; Low–Medium, 7–9; Medium, 10–12; Medium–High, 13–15. This is an author-developed assessment introduced during revision, not a validated or prospectively registered instrument; the underlying domains are reported explicitly so that each rating can be audited, and “in vitro only” or “not reported” is distinguished from demonstrated poor performance. The score measures the evidence available for a tissue, not the intrinsic quality of any construct. A domain that the included studies do not report scores 0, because no evidence exists to credit, and this is annotated in the cell; a 0 of this kind is not a finding of poor performance. Scores derive from the included studies only. This panel covers studies assigned to the implantation and regeneration pathway. Constructs intended as laboratory models are not scored here, because in vivo duration, in vivo functional outcome and animal-model relevance do not apply to them. * The endothelium is the only tissue for which a first-in-human trial has begun. That trial is company-sponsored and falls outside the inclusion criteria of this review; the published preclinical base for this layer remains the smallest of any corneal layer.

Tissue	Clinical Need	Examples of Bioprinted Constructs Reported	Translational Barriers	Preclinical Evidence (Studies; Highest Evaluation; Longest In Vivo Follow-Up)	Readiness Score (D1–D5; Total/15; Tier)
Corneal Epithelium	Limbal stem cell deficiency	LSC-laden scaffolds; GelMA-based (2025)	Immune rejection, stemness loss	9 regeneration studies; in vivo rat, rabbit, piglet; follow-up not consistently reported (≤4 wk); 1 limbal-niche model not scored	D1 2, D2 3, D3 2, D4 2, D5 3; total 12/15—Medium: functional repair in disease-matched large-eye models; follow-up ≤ 4 wk
Corneal Stroma	Stromal scarring or keratoconus	Multilayer collagen/GelMA scaffolds; dECM hybrids (2025)	Optical smoothness, biomechanics	34 regeneration studies; in vivo rabbit and rat, ex vivo porcine; longest follow-up 90 d; 4 in vitro models not scored	D1 3, D2 3, D3 3, D4 1, D5 2; total 12/15—Medium: largest evidence base and longest follow-up (90 d); no functional visual outcome
Endothelium	Graft shortage	iPSC-CEC constructs on DM substrates; hPSC-derived (2025)	Monolayer stability, pump function	3 studies; 1 in vivo rabbit (~4 wk); ex vivo rat, porcine, human Descemet’s membrane	D1 2, D2 2, D3 2, D4 2, D5 2; total 10/15—Medium *: in vivo clarity restoration on the smallest evidence base in this review
Conjunctiva	Symblepharon, scarring	Mucin-producing epithelial constructs; injectable micro-constructs (2025)	Goblet cell survival, inflammation	2 regeneration studies; in vivo rabbit, longest follow-up ~4 wk; 1 screening model not scored	D1 1, D2 3, D3 2, D4 1, D5 2; total 9/15—Low–Medium: two in vivo rabbit regeneration studies, longest follow-up ~4 wk; pterygium model not scored
Lens	Cataract/post-capsule fibrosis	PCO and lentoid models; GelMA-based PCO (2025)	Refractive index control	1 regeneration study; in vitro only; 1 screening model not scored	D1 1, D2 0, D3 0, D4 0, D5 0; total 1/15—Very Low: in vitro lens-epithelial prototype only; the PCO screening model is not scored
Sclera	Myopia, ectasia	Reinforcing hydrogel implants	Long-term IOP tolerance	1 study; in vivo guinea pig, follow-up 4 wk	D1 1, D2 2, D3 2, D4 2, D5 1; total 8/15—Low–Medium: axial elongation reduced and sclera thickened by 4 wk in a myopia model; single study in a rodent species
Lacrimal Gland	Dry eye	Organoid models, magnetic assembly; with gene therapy (2025)	Integration, vascularization	3 studies; in vitro only	Modeling pathway—not scored
Eyelid/Meibomian Gland	Meibomian gland dysfunction; tarsal plate loss	hADSC-laden atelocollagen/alginate tarsal plate scaffolds (2024)	Meibocyte differentiation, lipid secretion, lid biomechanics	1 study; in vivo rat, follow-up 4 wk	D1 1, D2 2, D3 2, D4 1, D5 1; total 7/15—Low–Medium: tarsal plate reconstruction at 4 wk; single study in rat

Abbreviations: CEC, corneal endothelial cell; dECM, decellularized extracellular matrix; DM, Descemet’s membrane; GelMA, gelatin methacryloyl; hADSC, human adipose-derived stem cell; hPSC, human pluripotent stem cell; IOP, intraocular pressure; iPSC, induced pluripotent stem cell; LSC, limbal epithelial stem cell; PCO, posterior capsule opacification.

**Table 4:** Proposed minimum reporting set for studies of bioprinted anterior segment ocular tissues. Items are grouped by reporting domain and reflect the parameters whose inconsistent reporting prevented quantitative synthesis in this review. The set is intended to complement, not replace, established guidelines for in vivo research (ARRIVE 2.0).

Reporting Domain	Minimum Items to Report (with Units and Conditions)
Construct and bioink	Full bioink composition with component concentrations (% *w*/*v*); crosslinker identity and crosslinking method; construct geometry and thickness (µm); cell type, source, passage number and seeding density (cells mL^−1^).
Printing process	Bioprinting modality; modality-specific parameters: for extrusion, nozzle diameter, pressure and printing speed; for light-based methods, light-source wavelength, intensity, exposure time and layer height; and a quantitative print-fidelity or resolution metric.
Cell viability	For cell-laden constructs: named viability assay; at least one immediate postprinting timepoint and one at day 7 or later; viability as a percentage with the denominator stated.
Optical transparency	For tissues where optical clarity is functionally relevant (e.g., cornea, lens): percentage transmittance at a stated wavelength or defined spectral range (nm); hydration state; presence or absence of cells; sample thickness.
Mechanical properties	Test modality named explicitly (compressive, tensile or storage modulus); value with SI units (kPa or MPa); hydration state; strain rate or oscillation frequency.
Tissue-specific function	At least one tissue-appropriate structural or functional readout (e.g., epithelial barrier integrity, keratocyte or endothelial marker expression, refractive or barrier function), with the assay named.
In vivo evaluation (where performed)	Species, animal model and defect type; number of animals; follow-up duration; adherence to the ARRIVE 2.0 guidelines [140].

## Data Availability

All data supporting this systematic review are derived from previously published studies cited within the manuscript. No new experimental data were generated. The review protocol was prospectively registered in PROSPERO (CRD420251113745).

## Supplementary Materials

The following supporting information can be downloaded at: https://www.mdpi.com/article/10.3390/cells15181686/s1, Table S1 (completed PRISMA 2020 checklist) and Table S2 (summary of the 61 included corneal studies, with extracted parameters). References [94,142,143,144,145,146,147,148,149,150] are cited in the Supplementary Materials.
