# Supplementary material for: 3D Bioprinting in Anterior Segment Ocular Tissue Engineering: Advances, Challenges, and Translational Pathways—A Systematic Review
Source: Cells. 2026 Sep 17;15(18):1686. doi: 10.3390/cells15181686 (PMC13605772; doi:10.3390/cells15181686)
Supplement: Supplementary file 1 [file cells-15-01686-s001.zip › cells-4549223-Supplementary Table S1.pdf]

PRISMA 2020 Checklist

Section and Topic	Item #	Checklist item	Location where item is reported
**TITLE**			
Title	1	Identify the report as a systematic review.	Title (p. 1). The report is identified as a systematic review in the title and in the first sentence of the Abstract.
**ABSTRACT**			
Abstract	2	See the PRISMA 2020 for Abstracts checklist.	Abstract (p. 1) and Highlights. Structured per the PRISMA 2020 for Abstracts checklist: objective, eligible designs, sources, synthesis approach, principal findings, limitations of the evidence and interpretation.
**INTRODUCTION**			
Rationale	3	Describe the rationale for the review in the context of existing knowledge.	Section 1.1 (Burden of Anterior Segment Disorders and the Promise of 3D Bioprinting), paragraphs 1–3, including what existing cornea-centred reviews do not cover.
Objectives	4	Provide an explicit statement of the objective(s) or question(s) the review addresses.	Section 1.1, final paragraph. Three explicit objectives: bioprinting maturity across the anterior segment; the tissue-specific factors explaining that heterogeneity; and the barriers to translation.
**METHODS**			
Eligibility criteria	5	Specify the inclusion and exclusion criteria for the review and how studies were grouped for the syntheses.	Section 2.2 (Study Selection). Inclusion and exclusion criteria, then three sequential eligibility tests (fabrication method; cellular component and intent; tissue specificity). Studies were grouped for synthesis by target tissue and, within the cornea, by layer (Sections 3.2–3.3).
Information sources	6	Specify all databases, registers, websites, organisations, reference lists and other sources searched or consulted to identify studies. Specify the date when each source was last searched or consulted.	Section 2.1 (Data Sources and Search Strategy). PubMed and Scopus, backward citation screening of all included full texts, and a search update adding adnexa-specific terms. Date of last search: August 2026 (Section 2.1; Figure 2). No registers, websites or organisations were searched (Figure 2).
Search strategy	7	Present the full search strategies for all databases, registers and websites, including any filters and limits used.	Section 2.1. The complete Boolean strategy is reproduced verbatim in the text, together with the English-language limit and the adnexa-specific terms added at the search update.
Selection process	8	Specify the methods used to decide whether a study met the inclusion criteria of the review, including how many reviewers screened each record and each report retrieved, whether they worked independently, and if applicable, details of automation tools used in the process.	Section 2.2. Duplicates removed in Zotero 7.0.24; two reviewers (K.T., M.S.) screened independently at title/abstract and full text, with disagreements resolved by a third reviewer (S.P.). No automation tools were used (Figure 2).
Data collection process	9	Specify the methods used to collect data from reports, including how many reviewers collected data from each report, whether they worked independently, any processes for obtaining or confirming data from study investigators, and if applicable, details of automation tools used in the process.	Section 2.3 (Data Extraction and Synthesis). Two reviewers (K.T., M.S.) extracted data independently using a standardized form. No automation tools were used and study investigators were not contacted.
Data items	10a	List and define all outcomes for which data were sought. Specify whether all results that were compatible with each outcome domain in each study were sought (e.g. for all measures, time points, analyses), and if not, the methods used to decide which results to collect.	Section 2.3. Outcome domains sought for all studies: cell viability (percentage with assay and timepoint); optical transparency (percentage with wavelength or wavelength range); biomechanical performance (modulus type named, value with units). All compatible results were sought and reported; outcomes not measured are recorded as N/R. Study-level values appear in Table 1 and Table S1.

PRISMA 2020 Checklist

Section and Topic	Item #	Checklist item	Location where item is reported
	10b	List and define all other variables for which data were sought (e.g. participant and intervention characteristics, funding sources). Describe any assumptions made about any missing or unclear information.	Section 2.3. Study identification (authors, year, journal), target tissue or corneal layer, application, bioink composition, crosslinking method, bioprinting modality, digital modelling workflow, cell type and source, printing parameters, and experimental model (in vitro, ex vivo, in vivo with species). Items not reported by the source are recorded as N/R.
Study risk of bias assessment	11	Specify the methods used to assess risk of bias in the included studies, including details of the tool(s) used, how many reviewers assessed each study and whether they worked independently, and if applicable, details of automation tools used in the process.	Section 2.4 (Appraisal of Methodological Quality). A formal risk-of-bias assessment was NOT performed. The rationale is stated: no single validated instrument covers the range of designs included, most of which are early-stage in vitro feasibility or material-characterization studies without standardized endpoints, and applying a tool to the in vivo subset alone would not be comparable across the review. Studies were instead appraised by two reviewers for reproducibility, translational relevance and reporting completeness.
Effect measures	12	Specify for each outcome the effect measure(s) (e.g. risk ratio, mean difference) used in the synthesis or presentation of results.	Not applicable. No effect measures were used. Outcomes are reported as author-reported values with their assay, timepoint, wavelength and units, and are not pooled (Sections 2.3, 2.4 and 3.4).
Synthesis methods	13a	Describe the processes used to decide which studies were eligible for each synthesis (e.g. tabulating the study intervention characteristics and comparing against the planned groups for each synthesis (item #5)).	Sections 2.2 and 2.3. Studies were assigned to syntheses by target tissue and, within the cornea, by layer (epithelium, stroma, endothelium, multi-layer and specialized constructs); see Sections 3.2–3.3, Table 1 and Table S1.
	13b	Describe any methods required to prepare the data for presentation or synthesis, such as handling of missing summary statistics, or data conversions.	Sections 2.3 and 2.4. A single reporting convention was applied to the three principal outcomes; qualitative descriptors were not carried into the tables; transparency was treated as quantitative only where a numeric percentage was reported together with a numeric wavelength or range; overlapping reports from one experimental cohort were cited together and counted as a single study.
	13c	Describe any methods used to tabulate or visually display results of individual studies and syntheses.	Section 2.3. Individual study results are tabulated in Table 1 (non-corneal) and Table S1 (corneal) and displayed graphically in Figure 3 (transmission plotted at the wavelength each study reported) and Figure 4 (publication year, evidence maturity, reporting completeness, bioprinting modality).
	13d	Describe any methods used to synthesize results and provide a rationale for the choice(s). If meta-analysis was performed, describe the model(s), method(s) to identify the presence and extent of statistical heterogeneity, and software package(s) used.	Sections 2.3 and 3.4. Quantitative meta-analysis was not feasible given the heterogeneity of study designs, bioprinting methods and outcome measures; results were synthesized narratively and summarized in comparative tables. The rationale is stated in Section 2.3 and quantified in Section 3.4.
	13e	Describe any methods used to explore possible causes of heterogeneity among study results (e.g. subgroup analysis, meta-regression).	Section 3.4. Sources of heterogeneity (assay, timepoint, wavelength, hydration state, presence of cells, construct thickness and cell density) were examined narratively and displayed in Figures 3 and 4c. No subgroup analysis or meta-regression was performed, as no meta-analysis was conducted.
	13f	Describe any sensitivity analyses conducted to assess robustness of the	Not applicable. No sensitivity analyses were conducted, as no

Section and Topic	Item #	Checklist item	Location where item is reported
		synthesized results.	statistical synthesis was performed (Sections 2.3 and 3.4).
Reporting bias assessment	14	Describe any methods used to assess risk of bias due to missing results in a synthesis (arising from reporting biases).	Not performed. Risk of bias due to missing results in a synthesis was not assessed, as no synthesis of effect estimates was undertaken. Incomplete outcome reporting across the included literature is instead quantified in Section 3.4 and Figure 4c and discussed in Sections 4.1, 4.7 and 4.8.
Certainty assessment	15	Describe any methods used to assess certainty (or confidence) in the body of evidence for an outcome.	Not performed. Certainty of evidence was not rated with GRADE or an equivalent instrument, as no pooled effect estimates were produced. The maturity and limitations of the evidence base are discussed in Sections 2.4, 4.1 and 4.8.
**RESULTS**			
Study selection	16a	Describe the results of the search and selection process, from the number of records identified in the search to the number of studies included in the review, ideally using a flow diagram.	Section 3.1 and Figure 2 (PRISMA 2020 flow diagram). Records identified (n = 415; PubMed n = 179, Scopus n = 236); duplicates removed (n = 121); records screened (n = 294); records excluded at title/abstract (n = 225); reports sought and assessed for eligibility (n = 69); reports excluded (n = 10); reports identified by citation searching (n = 13); reports included (n = 72); studies included (n = 71).
	16b	Cite studies that might appear to meet the inclusion criteria, but which were excluded, and explain why they were excluded.	Section 3.1 and Figure 2. The ten reports excluded at full-text assessment are reported with reasons: not ocular-tissue-specific (n = 5); no cellular component at any stage (n = 3); not cell-laden bioprinting (n = 1); printed output a device with a drug payload (n = 1).
Study characteristics	17	Cite each included study and present its characteristics.	Table S1 (all 61 included corneal studies, each cited by its main-list reference number, with layer, application, cell type and source, bioink and crosslinking, modality, outcomes and model) and Table 1 (all 10 included non-corneal studies). Narrative characteristics are given in Sections 3.2–3.3.
Risk of bias in studies	18	Present assessments of risk of bias for each included study.	Not applicable. Risk of bias was not assessed for individual studies; see Section 2.4 for the rationale and for the reproducibility, translational relevance and reporting-completeness appraisal applied instead.
Results of individual studies	19	For all outcomes, present, for each study: (a) summary statistics for each group (where appropriate) and (b) an effect estimate and its precision (e.g. confidence/credible interval), ideally using structured tables or plots.	Table S1 and Table 1 report, for each study, cell viability, optical transparency and mechanical/degradation outcomes with the assay, timepoint, wavelength and units used by the source. Figure 3 plots every extractable transmission value at its reported wavelength. Sections 3.2–3.3 describe individual study results narratively.
Results of syntheses	20a	For each synthesis, briefly summarise the characteristics and risk of bias among contributing studies.	Sections 3.2–3.3 (by tissue and corneal layer) and Section 3.4 (cross-study synthesis), with contributing study characteristics summarized in Figure 4a, 4b and 4d. Risk of bias among contributing studies was not assessed (Section 2.4).
	20b	Present results of all statistical syntheses conducted. If meta-analysis was done, present for each the summary estimate and its precision (e.g. confidence/credible interval) and measures of statistical heterogeneity. If comparing groups, describe	Not applicable. No statistical synthesis or meta-analysis was performed; the reasons are given in Sections 2.3 and 3.4. The narrative synthesis is presented in Section 3.4 and Figure 4.

Section and Topic	Item #	Checklist item	Location where item is reported
		the direction of the effect.	
	20c	Present results of all investigations of possible causes of heterogeneity among study results.	Section 3.4 and Figure 4c, with the dispersion of transmission values and of the wavelengths at which they were measured shown in Figure 3. Section 4.7 sets out the reporting heterogeneity and the proposed remedy (Table 3).
	20d	Present results of all sensitivity analyses conducted to assess the robustness of the synthesized results.	Not applicable. No sensitivity analyses were conducted.
Reporting biases	21	Present assessments of risk of bias due to missing results (arising from reporting biases) for each synthesis assessed.	Not assessed. No formal assessment of risk of bias due to missing results was performed; inconsistent reporting of blinding and randomization in the animal studies and the absence of appropriate in vitro controls are noted in Section 4.1, and the limitation is acknowledged in Section 4.8.
Certainty of evidence	22	Present assessments of certainty (or confidence) in the body of evidence for each outcome assessed.	Not assessed. Certainty of evidence was not formally rated. The immaturity of the evidence base (most studies published from 2024 onward; longest in vivo follow-up 90 days) is discussed in Sections 3.4, 4.1 and 4.8.
**DISCUSSION**			
Discussion	23a	Provide a general interpretation of the results in the context of other evidence.	Sections 4.1–4.6. Section 4.1 interprets the distribution of research effort against clinical need and against the first-in-human endothelial trial; Table 2 summarizes translational readiness across tissues.
	23b	Discuss any limitations of the evidence included in the review.	Sections 3.4, 4.1, 4.7 and 4.8. The evidence base is recent and largely in vitro (40 of 71 studies), confined to small-animal models, with no reported follow-up beyond 90 days, no non-human primate or human implantation, and inconsistent reporting of viability, transparency and mechanics.
	23c	Discuss any limitations of the review processes used.	Section 4.8 (Limitations of This Review). Two databases and an English-language restriction; no validated risk-of-bias instrument; meta-analysis precluded by heterogeneity; reliance on author-reported outcomes without access to raw data; work published after the search cut-off not represented.
	23d	Discuss implications of the results for practice, policy, and future research.	Sections 4.2–4.7 and Section 5. Includes the proposed minimum reporting set for future studies (Section 4.7 and Table 3), regulatory and manufacturing implications (Section 4.5), and equity, cost and access considerations (Section 4.6).
**OTHER INFORMATION**			
Registration and protocol	24a	Provide registration information for the review, including register name and registration number, or state that the review was not registered.	Section 2.1 and the Data Availability Statement. Registered prospectively in PROSPERO (International Prospective Register of Systematic Reviews), registration number CRD420251113745.
	24b	Indicate where the review protocol can be accessed, or state that a protocol was not prepared.	Section 2.1. The protocol is publicly accessible in the PROSPERO record (CRD420251113745). No separate protocol paper was published.
	24c	Describe and explain any amendments to information provided at registration or in	Section 2.1. One amendment to the registered strategy: at the

Section and Topic	Item #	Checklist item	Location where item is reported
		the protocol.	search update, adnexa-specific terms ("eyelid", "tarsal plate", "meibomian gland", "nasolacrimal", "canaliculus") were added and screened against the same eligibility criteria. Eligibility criteria and outcome definitions were not otherwise amended.
Support	25	Describe sources of financial or non-financial support for the review, and the role of the funders or sponsors in the review.	Funding statement. Supported in part by the Bascom Palmer Eye Institute NIH Center Core Grant P30EY014801 and by a Research to Prevent Blindness Unrestricted Award (GR004596). The funders had no role in the design, conduct, analysis or reporting of this review.
Competing interests	26	Declare any competing interests of review authors.	Conflicts of Interest statement. The authors declare no competing interests related to this work.
Availability of data, code and other materials	27	Report which of the following are publicly available and where they can be found: template data collection forms; data extracted from included studies; data used for all analyses; analytic code; any other materials used in the review.	Data Availability Statement and Supplementary Materials. The data-extraction table for the 61 included corneal studies is provided as Table S1; extractions for the 10 non-corneal studies are in Table 1. The data-collection form and extraction conventions are described in Sections 2.3 and 2.4. No new experimental data were generated and no analytic code was used; all data derive from the published reports cited in the reference list.

From: Page MJ, McKenzie JE, Bossuyt PM, Boutron I, Hoffmann TC, Mulrow CD, et al. The PRISMA 2020 statement: an updated guideline for reporting systematic reviews. BMJ 2021;372:n71. doi: 10.1136/bmj.n71.
This work is licensed under CC BY 4.0. To view a copy of this license, visit https://creativecommons.org/licenses/by/4.0/
