# Supplementary material for: 3D Bioprinting in Anterior Segment Ocular Tissue Engineering: Advances, Challenges, and Translational Pathways—A Systematic Review
Source: Cells. 2026 Sep 17;15(18):1686. doi: 10.3390/cells15181686 (PMC13605772; doi:10.3390/cells15181686)
Supplement: Supplementary file 1 [file cells-15-01686-s001.zip › cells-4549223-Supplementary Table S2.pdf]

Supplementary Material

Table S2. Characteristics and reported outcomes of the 61 included corneal studies

Studies published between 2016 and 2026 that met the eligibility criteria and targeted an individual corneal layer (epithelium, stroma, endothelium) or multiple layers. For each study the table gives the targeted layer, application, cell type and source, bioink composition with crosslinking method, bioprinting technique, reported outcomes, and experimental model. Outcomes are consolidated in a single column: V = cell viability, T = optical transparency, M = mechanical properties and degradation.

Reporting conventions. Values are given as reported by the source study. Cell viability is given as a percentage with the assay and timepoint; optical transparency as a percentage with the wavelength or wavelength range at which it was measured; mechanical performance with the modulus type named and the value in the units used by the source. Compressive, tensile, elastic, Young's and storage moduli measure different quantities and are not interchangeable, and values are therefore reported without pooling. Where a study characterized several formulations, timepoints or measurement conditions, each is given separately rather than merged into a single range. Where an outcome was assessed but the source did not state a numeric value, the entry records the assay and the conditions under which it was assessed. N/R indicates that the outcome was not assessed.

Multiple reports of the same study. Where one research group reported overlapping work from a single experimental cohort in more than one publication, the reports were treated as a single study and both citations are given in the reference column. Sixty-one corneal studies are reported here across sixty-two reports.

Reference numbering. Numbers in square brackets in the first column correspond to the numbered reference list of the main manuscript.

Reference (year)	Layer	Application	Cells (source)	Bioink (crosslinking)	Technique	Reported outcomes — viability | transparency | mechanics and degradation	Model
**A) EPITHELIUM**							
Wu et al. (2016) [35]	Epithelium	Controllable degradation	HCECs	Alg/Col/Gel (calcium ions)	Extrusion-based	**V:** 94.6 ± 2.5% (post-print)<br>**T:** 62.2 ± 8.4% (630 nm)<br>**M:** Degradation controlled by sodium citrate	In vitro
Zhang et al. (2019) [57]	Epithelium	Epithelial sheet fabrication	HCECs	Extrusion: Alg/Gel (UV sterilization); DLP: GelMA	Extrusion-based + DLP	**V:** >80% (post-print)<br>**T:** 85–94% (300–700 nm)<br>**M:** YM 0.2 MPa; TS 0.22 MPa; stable for 14 days	In vitro
Zhong et al. (2021a) [8]	Epithelium	Dual ECM scaffolds for limbal stem cells	LSCs (rabbit/human)	GelMA/HAGM (LAP photocrosslink)	DLP	**V:** GelMA 86.7 ± 1.6%; HAGM 92.1 ± 0.8% (day 7)<br>**T:** N/R<br>**M:** Stiffness modulated by light exposure	In vitro
Pietryga et al. (2024) [7]	Epithelium	Limbal stem cell deficiency treatment	LSCs (porcine)	Fibrin (thrombin, enzymatic)	Extrusion-based	**V:** 91.3 ± 4.6% (day 0); 91.1 ± 8.2% (day 7)<br>**T:** N/R<br>**M:** Enzymatic degradation (fibrin)	In vitro
Wang et al. (2024b) [56]	Epithelium	Corneal alkali burn repair	LSCs (rat)	Chitosan-based (thermosensitive)	4D bioprinting	**V:** >90%<br>**T:** N/R<br>**M:** Swelling ~1097%; 26% weight remaining (day 10)	In vivo (rat, alkali burn)
Xie et al. (2024) [58]	Epithelium	Corneal epithelial injury repair	CECs (rat, rabbit, human)	Culture medium + LBGP/thrombin/fibrinogen (enzymatic)	Pneumatic spray printing	**V:** 98.4 ± 0.31% (0 PSI) to 87.6 ± 1.3% (20 PSI), 10 min post-print<br>**T:** N/R<br>**M:** N/R	In vitro; in vivo (rat, mechanical injury)
Wang et al. (2024a) [27]	Epithelium	Diabetic corneal epithelial repair	LSCs (rabbit)	Chitosan/carboxymethyl chitosan (thermosensitive)	4D bioprinting	**V:** >90% (post-print)<br>**T:** N/R<br>**M:** Porous structure	In vitro; in vivo (diabetic rabbit)
Wang et al. (2025b) [59]	Epithelium	LSC sheets for limbal stem cell deficiency	LSCs (rabbit)	LSC spheroids + collagen membrane (N/R)	Auto-bioprinting (aspiration-based)	**V:** >90% (post-print)<br>**T:** N/R<br>**M:** Stable sheets for transplantation	In vivo (rabbit)
Kauppila et al. (2025) [61]	Epithelium	Compartmentalized limbal niche for disease modeling	hPSC-derived limbal stem cell subpopulations (human)	HA-based bioinks + PA gel scaffold (N/R)	Extrusion-based	**V:** >90% (1 week)<br>**T:** N/R<br>**M:** Native tissue-like mechanical properties	In vitro
Shang et al. (2025) [60]	Epithelium (limbal)	Alkali burn repair; limbal stem cell delivery	Limbal stem cells (porcine)	Chitosan–carboxymethyl chitosan thermosensitive hydrogel (4D-CTH)	4D printing (extrusion, thermoresponsive)	**V:** High (live/dead); % N/R<br>**T:** N/R<br>**M:** N/R	In vitro; in vivo (Bama piglet, alkali burn)

Reference (year)	Layer	Application	Cells (source)	Bioink (crosslinking)	Technique	Reported outcomes — viability ∣ transparency ∣ mechanics and degradation	Model
**B) STROMA**							
Isaacson et al. (2018) [31]	Stroma	Corneal stroma equivalent	Keratocytes (human)	Alg/Col-MA (CaCl2 + UV/LAP)	Extrusion-based (FRESH)	**V:** 92% (day 1); 83% (day 7)<br>**T:** N/R<br>**M:** Thickness ~600 μm; stable for 7 days	In vitro
Kim et al. (2018a, 2019a) [54,55]	Stroma	Cornea-specific bioink: transparency, safety and in vivo biocompatibility	Turbinate-derived MSCs (human)	Cornea-derived dECM (Co-dECM; thermal gelation)	Extrusion-based	**V:** N/R<br>**T:** >75% (300–700 nm, day 28)<br>**M:** Construct stable for 28 days	In vitro; in vivo (mouse, 8 weeks; rabbit, 4 weeks)
Kim et al. (2019b) [49]	Stroma	Shear-induced alignment of collagen fibrils	Turbinate-derived MSCs, keratocyte-differentiated (human)	Co-dECM, shear-aligned via nozzle gauge (thermal gelation)	Extrusion-based	**V:** N/R<br>**T:** Transmittance measured across 300–700 nm (day 28)<br>**M:** N/R	In vitro (28 days); in vivo (rabbit, 4 weeks, n = 10)
Kilic Bektas and Hasirci (2019) [62]	Stroma	Corneal stroma engineering	Keratocytes (human)	GelMA (UV photocrosslink)	Extrusion-based	**V:** 98% (live/dead, day 21)<br>**T:** >80% (700 nm)<br>**M:** Compressive modulus up to 20 kPa	In vitro
Park et al. (2019) [72]	Stroma	Biocompatibility of bioprinted decellularized collagen sheet	Turbinate-derived MSCs (human)	dECM (incubator crosslink)	Extrusion-based	**V:** N/R<br>**T:** N/R<br>**M:** Half thickness post-implant; biodegradation tolerated	In vivo (rabbit)
Duarte Campos et al. (2019) [38]	Stroma	Collagen-based stromal 3D models	Keratocytes (human)	Agarose/collagen (N/R)	Drop-on-demand	**V:** Live/dead (days 1, 7); % N/R<br>**T:** Qualitative (OCT optical density vs native cornea)<br>**M:** Compressive tangent modulus 18.1 ± 3.5 kPa (20% strain)	In vitro
Kutlehria et al. (2020) [63]	Stroma	High-throughput stromal equivalents for screening	Keratocytes (human)	Alg/Gel/Col I (calcium chloride)	Stereolithography + extrusion + micro-transfer molding	**V:** 96 ± 5% (day 1); 86 ± 2% (day 14)<br>**T:** 75–90% (400–700 nm)<br>**M:** Elastic modulus 42.03 ± 7 kPa; surface resolution 25 μm; RMS roughness 6.68 ± 0.6 nm	In vitro
Mahdavi et al. (2020) [50]	Stroma	Corneal stroma equivalent	Keratocytes (human)	GelMA (visible-light crosslink)	Visible-light stereolithography	**V:** 81.86% (day 1)<br>**T:** Center 78–90%; edge 80–95% (400–1000 nm)<br>**M:** Stable	In vitro
Ulag et al. (2020) [150]	Stroma	Artificial cornea for stromal transplantation	Adipose-derived MSCs (human)	PVA + chitosan composites (N/R)	Extrusion-based	**V:** MTT 91.5% (day 1), 118% (day 3), 120.6% (day 7), CS constructs<br>**T:** 61% (PVA); decreases with higher CS content (400–800 nm)<br>**M:** High tensile strength; stable	In vitro
Mörö et al. (2022) [64]	Stroma	Corneal stromal model with innervation	Adipose-derived MSCs and keratocytes (human); iPSC-neurons (human)	HA-CDH; HA-DA-CDH (HA-Ald crosslink)	Extrusion-based	**V:** >95% (7 days)<br>**T:** 71.9 ± 1.3% (400 nm) and 94.1 ± 1.5% (700 nm) at 1 h; refractive index 1.343 ± 0.00052<br>**M:** Stable to 14 days; compression modulus 10.3 kPa	In vitro; ex vivo (porcine, anterior lamellar keratoplasty)
Song et al. (2022) [118]	Stroma	Corneal patch for regeneration	Corneal stromal cells	High-concentration collagen type I + riboflavin (UV crosslink)	Spiral printing	**V:** >90% (week 3)<br>**T:** 90–96% (400–700 nm)<br>**M:** Stable integration; degradation over 4 weeks	In vitro
Kostenko et al. (2022) [142]	Stroma	Alginate-based bioinks for corneal engineering	Corneal stromal fibroblasts (human)	Alginate (CaSO4 pre-crosslink; CaCl2)	Extrusion-based	**V:** ~80–90% (day 7)<br>**T:** N/R<br>**M:** Stable (7-day in vitro testing only)	In vitro
Boix-Lemonche et al. (2023) [73]	Stroma	Intracorneal implantation of MSC-laden scaffolds by femtosecond-laser-assisted intrastromal keratoplasty	Adipose-, bone marrow- and corneal stroma-derived MSCs (human)	NC (80% nanocellulose/20% alginate); NC_Col (70/20/10% human Col I) (CaCl2 crosslink)	Extrusion-based	**V:** High (days 2, 7, 14)<br>**T:** 65.8–99.5% (400–700 nm)<br>**M:** N/R	In vitro; ex vivo (porcine)
Zhang et al. (2023) [24]	Stroma	Artificial corneal stroma for corneal substitutes	Corneal stromal fibroblasts (human)	CECM–GelMA (photocrosslink)	DLP	**V:** ~75.3% (day 1)<br>**T:** ~86% (>500 nm)<br>**M:** Tunable mechanical properties	In vitro
Jia et al. (2023) [19]	Stroma	Custom hydrogel lenticule for refractive correction via intrastromal keratoplasty	Corneal epithelial, stromal and endothelial cells	Poly-NAGA + GelMA (PNG) (photocrosslink)	DLP	**V:** >90%<br>**T:** >85% (630 nm); refractive index 1.37; refractive power 46 D (vs 43 D human cornea)	In vitro; in vivo (rabbit)

Reference (year)	Layer	Application	Cells (source)	Bioink (crosslinking)	Technique	Reported outcomes — viability | transparency | mechanics and degradation	Model
						**M:** Comparable to human cornea	
Xue et al. (2023) [70]	Stroma (lamellar)	Lamellar corneal substitute with natural curvature	Stromal fibroblasts (human)	GelMA (thermal/UV crosslink)	Hybrid (DLP + extrusion)	**V:** High (3D culture)<br>**T:** ~85% (400–700 nm)<br>**M:** Modulus close to native; swelling ~10%	In vitro; in vivo (rabbit, 2-month intrastromal implantation)
Ghosh et al. (2024a) [52]	Stroma	Resilient transparent cornea stroma equivalent	Limbal MSCs (human)	dECM + silk fibroin (Eosin Y/TEA/NVP, green light)	Extrusion-based	**V:** Encapsulated ~85–90%; top-seeded ~90–92% (days 7–14)<br>**T:** ~87–90% (700 nm)<br>**M:** YM ~0.5 MPa (compressive); ~66–70% strain; partial enzymatic degradation ~14 h	In vitro
Ghosh et al. (2024b) [119]	Stroma	Anisotropic functional corneal stroma	Limbal MSCs (human)	Central: DCM hydrogel; peripheral: DCM/SSF hybrid (85:15)	Extrusion-based (multi-material)	**V:** 92.86 ± 1.87% (live/dead, day 1)<br>**T:** 60% (700 nm, bilayer)<br>**M:** YM ~0.58 MPa; ~113 kPa compressive strength; ~96% weight loss in 12 h (enzymatic)	In vitro
Lee et al. (2024a) [65]	Stroma	Artificial cornea scaffold for transplantation	Corneal stromal cells (rabbit)	bHAMA/BP-T (photocrosslink)	DLP	**V:** 88% (BP); 83% (FBP); 77% (LAP)<br>**T:** >90% (400–800 nm)<br>**M:** Tensile strength 200.46 kPa; elongation 20%	In vitro
Puistola et al. (2024a) [143]	Stroma	Corneal stroma substitute for corneal blindness	Adipose-derived stem cells differentiated to keratocytes (human)	HA-based bioink with decellularized ECM	Extrusion-based	**V:** Live/dead (days 1, 7); % N/R<br>**T:** Qualitative (visual, day 10)<br>**M:** Storage modulus 138.21 ± 7.26 Pa; loss modulus 6.15 ± 1.11 Pa (1 Hz)	In vitro
Puistola et al. (2024b) [66]	Stroma	Artificial corneal stroma for transplantation	Adipose-derived stem cells (human)	HA-based bioinks, soft (cell-laden) and stiff (acellular): HA-ALD and HA-DA-CDH	Extrusion-based (multi-material)	**V:** >98% (live/dead, days 1 and 7)<br>**T:** Cell-laden composite 74–86% (day 1), 67–84% (day 7); acellular composite 76–87% (day 1), 70–87% (day 7), all at 400–700 nm<br>**M:** Storage modulus ~60 → ~141 Pa over 14 days; low swelling	In vitro
Vijayaraghavan et al. (2024a) [144]	Stroma	Stromal regeneration	Keratocytes (goat)	GelMA/agarose (photocrosslink)	Extrusion-based	**V:** High<br>**T:** 60% (400–700 nm, day 7)<br>**M:** GelMA10/A0.5 ~75 μm pores, 14% weight loss (28 days); GelMA10/A1 ~55 μm pores, 9% weight loss	In vitro
Zhang et al. (2024) [67]	Stroma	Bioartificial cornea for lamellar keratoplasty	Corneal fibroblasts (rabbit, SMILE-derived)	Micro-nanofibrous dECM + photocurable component (photocrosslink)	DLP + electrospinning	**V:** >95% (live/dead, 14 days)<br>**T:** ≈95% plateau across 350–800 nm<br>**M:** Compressive modulus 56.91 ± 3.68 → 248.5 ± 35.67 kPa with 2% eDCSM; Young's modulus (0–20% strain) 19.56 ± 5.90 → 42.70 ± 3.93 kPa	In vitro; in vivo (rabbit)
Vijayaraghavan et al. (2024b) [145]	Stroma	Corneal stromal regeneration	Corneal stromal cells (goat)	GelMA/methylcellulose (UV photocrosslink)	Extrusion-based	**V:** High (14 days)<br>**T:** ≈78% (700 nm)<br>**M:** Compressive strength ≈54 kPa (GelMA5/MC8), ≈69 kPa (GelMA7/MC8); 15–19% weight loss over 30 days	In vitro
Khoshnood et al. (2024) [94]	Stroma	Ocular drug delivery and corneal tissue engineering	Corneal fibroblasts (mouse)	Gellan gum + polyethyleneimine + betamethasone sodium phosphate (citric acid ionic crosslink)	Extrusion-based	**V:** >90% (day 3)<br>**T:** ~80% (700 nm)<br>**M:** Adequate strength; partial degradation over 14 days; stable controlled release	In vitro
Brunel et al. (2025) [68]	Stroma	In situ delivery of stromal stem cells to partial-thickness wounds	Corneal stromal cells (human)	Collagen-based UNION (SPAAC covalent crosslink)	In situ deposition	**V:** >95% (live/dead)<br>**T:** >90% (550 nm, 14 days)<br>**M:** Storage modulus plateau ≈700 Pa; gelation crossover ≈10 min	In vitro; in vivo (rabbit)
Chand et al. (2025) [69]	Stroma	Biomimetic corneal stroma via DLP	Keratocytes (human)	GelMA + oxidized carboxymethyl cellulose (photocrosslink)	DLP	**V:** >93% (live/dead, days 1, 3 and 7)<br>**T:** 75.0 ± 1.2% (400 nm), 85.3 ± 0.6% (500 nm), 89.3 ± 0.4% (600 nm), 91.0 ± 0.3% (700 nm)<br>**M:** Compressive modulus 106.3 ± 7.7 kPa (native cornea 115.3 ± 13.6 kPa in the same study); central thickness 478.9 ± 56.5 μm, peripheral 864.0 ± 79.3 μm	In vitro
Usseglio et al. (2025) [71]	Stroma	Photochemical crosslinking with hand-held biopen	Corneal keratocytes (human)	GelMA/PEGDA/PI (photochemical, UV)	Biopen bioprinting	**V: Live/dead (hCK); % N/R**<br>**T: N/R**	Ex vivo (porcine cornea)

Reference (year)	Layer	Application	Cells (source)	Bioink (crosslinking)	Technique	Reported outcomes — viability | transparency | mechanics and degradation	Model
						**M: Elastic modulus 22 ± 1.0 kPa (lowest formulation)**	
Yoon et al. (2025) [76]	Stroma	Electron-beam sterilization of bioprinted stromal patches	Keratocytes (human)	Co-dECM (thermal gelation, 37 °C, 30 min)	Extrusion-based	**V:** 90.20 ± 3.30% (EB-sterilized) and 79.90 ± 14.60% (pre-sterilized), live/dead, day 17<br>**T:** Transmittance measured at 550 nm and across 300–800 nm (day 17)<br>**M:** Compressive modulus measured (0–5% strain); patch thickness ~500–750 µm	In vitro
Choi et al. (2025) [32]	Stroma	3D corneal model using collagen bioink	Corneal stromal cells (human)	Collagen I (thermal gelation)	Extrusion-based	**V:** High (4 weeks)<br>**T:** N/R<br>**M:** Water retention similar to native tissue; stable	In vitro
Xu et al. (2025) [33]	Stroma	GelMA/collagen hydrogel for stromal regeneration	Corneal stromal cells (human)	GelMA/collagen (UV photocrosslink)	Extrusion-based	**V:** >90% (post-print)<br>**T:** >85% (400–700 nm)<br>**M:** Modulus ~0.1 MPa; stable	In vitro; in vivo (rabbit)
Gao et al. (2025) [39]	Stroma	Effect of print parameters on hydrogel transparency	N/R (acellular focus; cell-seeding assays)	Collagen (N/R)	Extrusion-based	**V:** ≈90% (day 0); >90% (L-929, day 12)<br>**T:** 89.8% (700 nm, optimized); 48.4% at 4% collagen<br>**M:** N/R	In vitro
Basoz et al. (2025) [74]	Stroma	Corneal stroma substitute	Keratocytes (human)	GelMA (photocrosslink)	Extrusion-based	**V:** Live/dead (days 1–21); % N/R<br>**T:** ≈60% (400 nm); ≈70% (700 nm)<br>**M:** Compressive modulus ≈136 kPa (cell-free); ≈281 kPa (cell-loaded)	In vitro; in vivo (rabbit, 90 days)
Taoum et al. (2025) [146]	Stroma	Keratocyte-lineage cell sourcing for corneal bioprinting	Bone marrow-derived MSCs differentiated to keratocyte-like cells (human)	0.5% agarose + 0.2% collagen type I (thermal gelation)	Drop-on-demand	**V:** N/R<br>**T:** N/R<br>**M:** N/R	In vitro (21 days)
Marin-Tapia et al. (2025) [147]	Stroma	Scaffold-free corneal construct	Placenta-derived mesenchymal stem cells (human)	Decellularized corneal ECM + sodium alginate + type B gelatin (CaCl2 ionic crosslink)	Extrusion (scaffold-free)	**V:** ≈95% (day 1); ≈83% (day 7)<br>**T:** >80% (400–700 nm)<br>**M:** Young's modulus (AFM) comparable to native cornea	In vitro (14 days)
Balters and Reichl (2026) [148]	Stroma	Keratin-based bioink for stromal constructs	Immortalized human corneal keratocytes and corneal fibroblasts	HAMA ± collagen or keratin (aqueous/alkaline); 405 nm UV	Extrusion-based	**V:** Live/dead (days 0–28)<br>**T:** >80% at 300–800 nm (day 28); ≈60% at 300–800 nm (day 0, aqueous keratin)<br>**M:** Compressive modulus ≈2–6 kPa (28 days)	In vitro (4 weeks)
Vijayaraghavan et al. (2026) [149]	Stroma	Stromal keratocyte phenotype maintenance	Corneal stromal cells (goat)	GelMA + carboxymethyl chitosan (light-activated crosslink)	Extrusion-based	**V:** Viable (live/dead and MTT)<br>**T:** Up to 80% at 14 days<br>**M:** Enhanced thermal stability; degradation and swelling characterized	In vitro (14 days)
Kethiri et al. (2026) [75]	Stroma	Injected and bioprinted HA-based constructs delivered to corneal stromal pockets	hASC-derived corneal stromal keratocytes (human)	Hyaluronic acid-based bioink (in situ gelation)	Extrusion-based	**V:** High (live/dead, in vitro)<br>**T:** Maintained (qualitative)<br>**M:** Stable in situ gelation; integration with host stroma	In vitro; in vivo (rat, stromal pocket)
**C) ENDOTHELIUM**							
Kim et al. (2018b) [77]	Endothelium	Bioprinted endothelium with RNase 5-overexpressing HCECs	Corneal endothelial cells (human, RNase 5-overexpressing)	Gelatin-based bioink on lyophilized amniotic membrane	Extrusion-based	**V:** N/R<br>**T:** Qualitative (clinical clarity in vivo)<br>**M:** N/R	In vivo (rabbit)
Grönroos et al. (2024) [37]	Endothelium	Bioprinted corneal endothelium for transplantation	hPSC-derived corneal endothelial cells (human)	Hydrazone-crosslinked hyaluronic acid	Extrusion-based	**V:** ~92.5% (day 7; no difference from 2D controls)<br>**T:** N/R<br>**M:** 26 ± 5% of original weight remaining by day 10; shape fidelity to 7 days	In vitro; ex vivo (rat, porcine, human corneas)
Overmass et al. (2024) [78]	Endothelium	Bioengineered Descemet's membrane for endothelial transplantation	Corneal endothelial cells (human)	Collagen type IV (photocrosslink)	Extrusion-based	**V:** High cytocompatibility (adhesion, confluence)<br>**T:** 97.8 ± 0.5% (400–700 nm)<br>**M:** Young's modulus 89.5 ± 23.9 kPa (tensile); 49 ± 14 kPa (compressive)	In vitro
**D) MULTI-LAYER, FULL-THICKNESS AND SPECIALIZED CONSTRUCTS**							

Reference (year)	Layer	Application	Cells (source)	Bioink (crosslinking)	Technique	Reported outcomes — viability | transparency | mechanics and degradation	Model
Sorkio et al. (2018) [28]	Epithelium and stroma	Native-like 3D corneal structures from human stem cells	ESC-derived limbal epithelial stem cells; adipose stem cells (human)	Laminin/collagen I (N/R)	Laser-assisted bioprinting (LaBP/LIFT)	**V:** Live/dead and PrestoBlue (days 1–7); % N/R<br>**T:** N/R<br>**M:** Stable integration	Ex vivo (porcine corneal organ culture)
He et al. (2022) [30]	Epithelium and stroma	Epithelium/stroma bilayer implant for corneal regeneration	Corneal epithelial cells and adipose-derived MSCs (rabbit)	GelMA/PEGDA (photocrosslink)	DLP	**V:** ~90% (live/dead, day 7)<br>**T:** 90.7% (PEGDA-GelMA-10-5), 86.3% (15-5), 82.5% (20-5), all at 600 nm; native cornea 77.9% at 600 nm in the same study<br>**M:** Compressive modulus ~100.7 kPa; tensile strength ~0.56 MPa; swelling ~100%; degrades in ~28 days (collagenase)	In vitro; in vivo (rabbit, anterior lamellar keratoplasty)
Puistola et al. (2025) [81]	Epithelium and stroma	Multi-material stem cell-based cornea with heterogeneous design	ASC-derived keratocytes (stroma); iPSC-derived limbal epithelial stem cells (epithelium)	Hyaluronic acid-based, varying stiffness (hydrazone crosslink)	Extrusion-based (multi-material)	**V:** Live/dead (days 1 and 7)<br>**T:** Transmittance measured across 400–700 nm<br>**M:** Storage modulus 112.2 ± 6.6 Pa; loss modulus 20.5 ± 3.3 Pa	In vitro
You et al. (2022) [84]	Full thickness (perforation repair)	In situ sealing of full-thickness corneal perforations	None (acellular adhesive)	hPL-based fibrin bio-adhesive (thrombin, enzymatic)	Extrusion-based (handheld, in situ)	**V:** Not applicable (acellular)<br>**T:** N/R<br>**M:** Storage modulus during gelation; adhesiveness reported	In vitro; in vivo (rabbit, n = 5)
Chaudhary et al. (2024) [82]	Full thickness	Artificial cornea with aligned fibrous structure	Acellular scaffold (cells seeded post-print)	Sodium alginate + cellulose nanofibers (poly-L-lysine-co-L-glutamic acid + CaCl2)	Extrusion-based	**V:** 82–96% (alamarBlue, day 7; three formulations)<br>**T:** 86% (550 nm); >82% (400–700 nm)<br>**M:** Compressive modulus up to 2360 kPa	In vitro
Ghosh et al. (2025) [85]	Full thickness (patch)	Adhesive corneal patch for sutureless repair	N/R (acellular)	Dopamine-conjugated silk fibroin + dECM (photocrosslink)	Extrusion-based (multi-material)	**V:** >90% (DCM, DCM/SF); >85% (silk), day 14<br>**T:** 90% (700 nm, DCM/SF); 80% (dual-crosslinked DCM)<br>**M:** Adhesion ~85 kPa; modulus ~0.48 MPa; stable	In vitro; ex vivo
Bhutani et al. (2024) [83]	Stroma (lenticule)	Biopolymeric corneal lenticules as transplant substitute	Corneal stromal cells (human cadaveric)	HA-MA/GelMA (photocrosslink)	DLP	**V:** >97% (3 weeks)<br>**T:** 85.45 ± 0.14% (400–700 nm)<br>**M:** Compressive modulus 535.42 ± 29.05 kPa; adhesive strength 58.67 ± 17.5 kPa	In vitro
Nie et al. (2024) [80]	Multi-layer (epithelium and stroma)	Corneal regeneration; growth factor and drug delivery	Acellular at implantation; rCECs, rASCs, L929 in vitro	Gelatin + carbohydrazide-modified alginate (Ca2+ then EDC), with rhEGF and trichostatin A	Extrusion (multi-nozzle)	**V:** N/R<br>**T:** 81.5 ± 1.2%; 82.8 ± 1.3% (500 nm)<br>**M:** Tensile strength 1.068 MPa; toughness 677.6 kJ/m²; elongation >400%; suturable	In vitro; in vivo (rabbit, anterior lamellar keratoplasty)
Wang et al. (2025a) [29]	Multi-layer (limbus: epithelial and niche layers)	Limbal stem cell deficiency; biomimetic limbal implant	Corneal epithelial cells and corneal stromal stem cells (human)	GelMA + PEGDA (DLP photocrosslink)	DLP	**V:** Maintained (live/dead); % N/R<br>**T:** 93% (600 nm)<br>**M:** Tensile and compressive modulus (10–20% strain)	In vitro; in vivo (rabbit LSCD, 4 weeks)
Nie et al. (2026) [79]	Multi-layer (epithelium and stroma)	Suture-free corneal regeneration	Corneal epithelial cells and corneal stromal stem cells	GelMA + 2-aminoethyl methacrylated chondroitin sulfate (CSAMA); in situ photocrosslinkable bioadhesive	DLP	**V:** High (live/dead, 7 days); % N/R<br>**T:** 94.1 ± 0.5% (600 nm)<br>**M:** Tensile strength 61.6 ± 1.9 kPa; elastic modulus 47.5 ± 2.5 kPa; elongation 111.2 ± 8.1%	In vitro; in vivo (rabbit ALK, 4 weeks)

Abbreviations: 4D printing, stimulus-responsive (four-dimensional) printing; 4D-CTH, chitosan–carboxymethyl chitosan thermosensitive hydrogel; AFM, atomic force microscopy; Alg, alginate; ALK, anterior lamellar keratoplasty; ASC / hASC / rASC, adipose-derived stem cell (human / rat); bHAMA, bio-orthogonally modified methacrylated hyaluronic acid; BP / BP-T / FBP, benzophenone-based photoinitiators (thiolated / functionalized); CEC, corneal epithelial cell; CECM, corneal extracellular matrix; Co-dECM, cornea-derived decellularized extracellular matrix; Col, collagen; CS, chitosan; CSAMA, 2-aminoethyl methacrylated chondroitin sulfate; DCM, decellularized corneal matrix; dECM, decellularized extracellular matrix; DLP, digital light processing; EB, electron beam (sterilization); ECM, extracellular matrix; EDC, 1-ethyl-3-(3-dimethylaminopropyl)carbodiimide; eDCSM, electrospun decellularized corneal stromal matrix; ESC, embryonic stem cell; FRESH, freeform reversible embedding of suspended hydrogels; GelMA, gelatin methacryloyl; HA, hyaluronic acid; HA-Ald, aldehyde-modified hyaluronic acid; HA-CDH, carbodihydrazide-modified hyaluronic acid; HA-DA, dopamine-conjugated hyaluronic acid; HAMA / HA-MA / HAGM, methacrylated hyaluronic acid; HCEC, human corneal epithelial cell; hCK, human corneal keratocyte; hPL, human platelet lysate; hPSC, human pluripotent stem cell; iPSC, induced pluripotent stem cell; L929, murine fibroblast cell line; LaBP, laser-assisted bioprinting; LAP, lithium phenyl-2,4,6-trimethylbenzoylphosphinate (photoinitiator); LBGP, Lycium barbarum glycopeptide; LIFT, laser-induced forward transfer; LSC, limbal stem cell; LSCD, limbal stem cell deficiency; MC, methylcellulose; MSC, mesenchymal stem cell; MTT, 3-(4,5-dimethylthiazol-2-yl)-2,5-diphenyltetrazolium bromide assay; N/R, not reported; NAGA, N-acryloyl glycinamide; NC, nanocellulose; NVP, N-vinylpyrrolidone (co-monomer); OCT, optical coherence tomography; PA, polyacrylamide; PEGDA, poly(ethylene glycol) diacrylate; PNG, poly(NAGA)–GelMA composite; PSI, pounds per square inch (spray pressure); PVA, poly(vinyl alcohol); rCEC, rabbit corneal epithelial cell; rhEGF, recombinant human epidermal growth factor; RMS, root mean square (surface roughness); SF, silk fibroin; SMILE, small-incision lenticule extraction; SPAAC, strain-promoted azide–alkyne cycloaddition; SSF, sericin-free silk fibroin; TEA, triethanolamine (co-initiator); TS, tensile strength; UV, ultraviolet; YM, Young’s modulus.
